# Modeling Co-Expression across Species for Complex Traits: Insights to the Difference of Human and Mouse Embryonic Stem Cells

**DOI:** 10.1371/journal.pcbi.1000707

**Published:** 2010-03-12

**Authors:** Jun Cai, Dan Xie, Zhewen Fan, Hiram Chipperfield, John Marden, Wing H. Wong, Sheng Zhong

**Affiliations:** 1 Department of Bioengineering, University of Illinois at Urbana Champaign, Urbana, Illinois, United States of America; 2 Department of Statistics, University of Illinois at Urbana Champaign, Urbana, Illinois, United States of America; 3 Department of Statistics, Stanford University, Stanford, California, United States of America; 4 Department of Health Research and Policy, Stanford University, Stanford, California, United States of America; 5 Institute of Medical Biology, Singapore; 6 Institute of Genomic Biology, University of Illinois at Urbana Champaign, Urbana, Illinois, United States of America; King's College London, United Kingdom

## Abstract

Complex interactions between genes or proteins contribute substantially to phenotypic evolution. We present a probabilistic model and a maximum likelihood approach for cross-species clustering analysis and for identification of conserved as well as species-specific co-expression modules. This model enables a “soft” cross-species clustering (SCSC) approach by encouraging but not enforcing orthologous genes to be grouped into the same cluster. SCSC is therefore robust to obscure orthologous relationships and can reflect different functional roles of orthologous genes in different species. We generated a time-course gene expression dataset for differentiating mouse embryonic stem (ES) cells, and compiled a dataset of published gene expression data on differentiating human ES cells. Applying SCSC to analyze these datasets, we identified conserved and species-specific gene regulatory modules. Together with protein-DNA binding data, an SCSC cluster specifically induced in murine ES cells indicated that the KLF2/4/5 transcription factors, although critical to maintaining the pluripotent phenotype in mouse ES cells, were decoupled from the OCT4/SOX2/NANOG regulatory module in human ES cells. Two of the target genes of murine KLF2/4/5, *LIN28* and *NODAL*, were rewired to be targets of OCT4/SOX2/NANOG in human ES cells. Moreover, by mapping SCSC clusters onto KEGG signaling pathways, we identified the signal transduction components that were induced in pluripotent ES cells in either a conserved or a species-specific manner. These results suggest that the pluripotent cell identity can be established and maintained through more than one gene regulatory network.

## Author Summary

A major goal in biology is to understand the evolution of complex traits, such as the development of multicellular body plans. To a certain extent, complex traits are governed by regulated gene expression. The comparison expression data between species requires extra considerations than sequence comparison, because gene expression is not static and the level of expression is influenced by external conditions. Considering that co-expression patterns are often comparable across species, we developed a statistical model for cross-species clustering analysis. The model allows each species to create its own clusters of the genes but also encourages the species to borrow strength from each others' clusters of orthologous genes. The result is a pairing of clusters, one from each species, where the paired clusters share many but not necessarily all orthologous genes. The model-based approach not only reduces subjective influence but also enables effective use of evolutionary dependence. Applying this model to analyze human and mouse embryonic stem (ES) cell data, we identified the transcription factors and the signaling proteins that are specifically expressed in either human or mouse ES cells. These results suggest that the pluripotent cell identity can be established and maintained through more than one gene regulatory network.

## Introduction

A major goal in biology is to understand the evolution of complex traits, such as the development of multicellular body plans or an organism's physical state as it ages [1]. To a certain extent, complex traits are governed by regulated gene expression, and considerable plasticity exists such that the same or a similar phenotypic outcome may arise from the same or vastly different gene regulatory programs across species [1],[2]. Methods for identifying evolutionarily conserved as well as alternative gene regulatory pathways underlying a biological trait should enable deeper mechanistic understanding of the processes that shaped the trait.

Cross-species comparative analyses have made fundamental contributions to biology, most remarkably exhibited by comparative analysis of genomic sequences [2]. With the growing availability of functional genomic data, comparative paradigms are now being extended to the study of other functional attributes, most notably gene expression (e.g., [3],[4],[5],[6],[7] reviewed in [8]). Major advantages of gene expression comparison include but are not limited to pinpointing the genes and tissues whose expression tends to evolve at an accelerated or reduced rate [4],[9], improving functional gene annotation [3], discovering conserved genetic modules and pathways [5],[7] and tracing phenotypic diversity by differential expression of specific regulatory genes [3],[4]. More recently, cross-species expression data have been used for inferring the evolution of interaction and regulatory networks [4],[10].

A major challenge in comparing expression data between organisms is that gene expression is not static and the level of expression is influenced by external conditions. This difficulty was circumvented in the special cases in which identical perturbations could be applied across species, as in comparisons of the sexes across species [11]. In the absence of identical perturbations, the co-expression between gene pairs remains comparable across species [8]. Therefore co-expression based analysis has been widely applied to compare gene expression datasets across phylogenetically close [3],[10] and distant species [5],[7],[12]. These efforts often focused on identifying conserved co-expression modules, groups of genes whose expression profiles were correlated in multiple species. Because the co-regulatory relationship of these genes was conserved, they were considered to function in a coordinated manner. The methods to identify these modules were based on first applying preset thresholds to expression correlation in each species and then intersecting the groups of orthologous genes across species [5],[12],[13],[14],[15],[16]. Such approaches were straightforward but often strongly influenced by subjective inputs from the researcher, for example, in the choice of correlation thresholds. An exception to the ad hoc thresholds was that Ramani et al. used known interaction proteins to train a threshold of co-expression [16]. This approach worked for protein-protein interaction analysis, but would require a lot empirical data to train similar thresholds for the analyses of other regulatory relationships, such as the relationships between transcription factors (TFs) and their target genes. Another limitation of the methods discussed above is that these methods do not uncover species-specific co-expression patterns, which may be important for explaining and understanding the evolution of novel features, e.g., the unique liver genes in human as opposed to other primates [17].

Automatic clustering algorithms, such as K-means and hierarchical clustering, have been widely used in gene expression data analysis to discover co-expression patterns that can be translated to biological knowledge or new hypotheses [18]. It is thus a natural step to extend these algorithms to cross-species analysis. However, clustering remains a difficult problem, as exemplified by ad hoc criteria for choosing optimal clusters and results being sensitive to the initial conditions. Naively applying these algorithms to multiple species, for example, by clustering each species separately and then combining the clustering results, will likely amplify the computational errors made in each species. A better approach is to customize the clustering methods for cross-species analysis, taking advantage of the evolutionary context to minimize clustering errors. Two methods, DCA [3] and CoherentCluster [7], were proposed recently in this direction. However, these two methods lacked statistical models and did not maximize the use of data. For example, the expression of one species was used for constraining the clustering of the other species, but not vice versa. Ideally, some iterative schemes, such as are common for many machine learning algorithms, would be implemented to simultaneously cluster genes in multiple species.

We have developed a statistical model for cross-species clustering analysis. The model allows each species to create its own clusters of the genes but also encourages the species to borrow strength from each others' clusters of orthologous genes. The result is a pairing of clusters, one from each species, where the paired clusters share many but not necessarily all orthologous genes. The clustering and degree of overlap are chosen by the data through maximum likelihood estimation. The model-based approach not only reduces subjective influence but also enables effective use of evolutionary dependence.

## Results

### Model based soft cross-species clustering

A model-based Soft Cross-Species Clustering (SCSC) method was developed. The rationale of this model stems from the following observations and intuitions. First, clusters of co-expressed genes may be conserved across a large evolutionary distance, in the sense that the orthologous genes also exhibit correlated expression [19]. Empirically, this is consistent with the observation that shared cis-regulatory elements and cis-regulatory modules that regulate a set of co-expressed genes in one species are often found to be enriched in the regulatory regions of the orthologous sets of genes in other species [20]. The conservation of clusters also makes evolutionary sense because a cluster may correspond to a regulatory program that is functionally important and thus resistant to change [21]. Second, rewiring across clusters, i.e., the change of cluster membership of orthologous genes, is also observed in phylogenetically related species [22],[23]. This rewiring process can reflect simple sequence changes such as gain or loss of transcription factor binding sites. The difference of regulatory programs across species is believed to be an important source of evolutionary diversity or novelty [17],[22],[23]. Finally, the expression patterns of orthologous clusters may not be conserved, reflecting either a change in the activities of the trans-acting factors (thus all the genes in a cluster will change their expression pattern, but their co-regulatory relationship is maintained) [22] or differences in experimental conditions across species.

**Figure 1:**
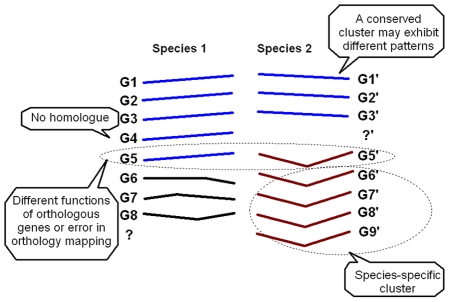
Major features and assumptions of the SCSC method. 1. G1–G8 are eight genes in Species 1, which have orthologues G1′–G8′ in Species 2. 2. G4 and G9′ do not have orthologues, but they participate in the clustering analysis. 3. The shapes of the lines represent gene expression patterns. For example, G1 has an increasing pattern and G6′ has a first decreasing and then increasing pattern. 4. The genes with the same color, except for the black color, are clustered together. Genes in black are “scattered” genes, which form a singleton cluster each.

We formulated the above observations and intuitions into a probabilistic model, with certain simplifications that made the model mathematically tractable. First, we assumed that in every species there are a certain number of clusters that can be mapped one-to-one (called orthologous clusters), with each cluster corresponding to an essential regulatory program. However, the mean profiles of orthologous clusters were assumed to be independent. Second, the expression of a gene in a cluster was assumed to be a sample from a Gaussian distribution, which was the characteristic or mean profile of this cluster. This assumption is commonly made in model-based clustering analysis [18]. Third, a gene tends to belong to the orthologous clusters in the two species. In other words, the prior probability that a gene belongs to the clusters (i, i′) where i and i′ are the indices of the corresponding clusters in two species respectively, was larger than that for non-orthologous clusters. This intuition was formally represented by a logistic regression of prior probabilities over the cluster indices (see Methods). Overall, the model captured the main observations discussed above: that cluster structure tends to be conserved, that change of cluster membership should be allowed (as G5′ in Figure 1), and that the mean expression profiles of orthologous clusters are relatively independent.

### Synthetic data

The performance of SCSC was compared with that of DCA, K-means, hierarchical clustering, MCLUST, WGCNA and CLICK clustering [24] on six synthetic datasets (Table S1, Text S1). Because the performance of K-means, hierarchical clustering, MCLUST, WGCNA and CLICK algorithms were optimized within each species, if the information of conservation of co-clustering did not help, they should outperform SCSC and DCA. In four of the six synthetic datasets, CLICK and SCSC outperformed the other algorithms on the center-scatter score (Figure S1), which is consistent to CLICK's capability of filtering out singleton genes and identifying very tight clusters. In all the rest comparisons, SCSC outperformed K-means, hierarchical clustering MCLUST, WGCNA, and CLICK, which in turn outperformed DCA (Figure S1). These results suggest that although conserved co-clustering information could help to improve clustering performance, the power of such information is released in a model-based approach (SCSC) but shackled in a heuristic algorithm (DCA). DCA essentially sequentially performs two hierarchical clustering in the two species, with no iteration.

### Evaluation on synthetic data with errors in the orthology map

To mimic errors in the orthology map or the scenario where some orthologous genes have divergent functional roles in two species, we permuted a proportion (10%–30%) of the orthologous relations into wrong matches in the first synthetic dataset. SCSC, DCA, and K-means were executed on these datasets with orthology errors (Table S2). As the proportion of misplaced orthology links increased, all four algorithms showed decreased performance as expected. However, SCSC demonstrated robustness against orthology errors in that its performance on the dataset with 30% orthology errors was better than those of the other three algorithms under 0% orthology errors.

### Embryonic stem cell data

The biological process that inspired the SCSC model is cellular differentiation, a fundamental process occurring universally in multicellular organisms. Embryonic stem (ES) cells were used as a tool to study this process. ES cells are characterized by the ability to self-renew and differentiate into every cell type found in the mature organism. We are interested in determining the extent to which molecular circuits that underlie ES cell phenotypes and the processes of commitment and differentiation are conserved across species.

Human and mouse ES (hES and mES) cells share the critical properties of ES cells but do not employ the identical set of transcription factors. For example, transcription factor FOXD3 is required for mES cell self-renewal [25], but its expression appears to be non-essential for hES [26]. Similarly, STAT3, a transcription factor downstream to LIF signaling, is also required for self-renewal and the maintenance of pluripotency of mES cells, but it seems to be dispensable in hES cells [27]. *We hypothesize that the pluripotent cell identity can be established and maintained through more than one gene regulatory network*. These regulatory networks share core components that are universally indispensable for pluripotency. Peripheral components, though critical for cell fate, can be implemented using alternative designs. If this hypothesis is verified, the conserved and species-specific ES cell gene clusters may reveal the essential and peripheral components of gene regulatory networks underlining pluripotency, which may in turn assist the search for gene sets that are capable of reprogramming adult cells into a pluripotent state with higher efficiency [28],[29].

**Figure 2:**
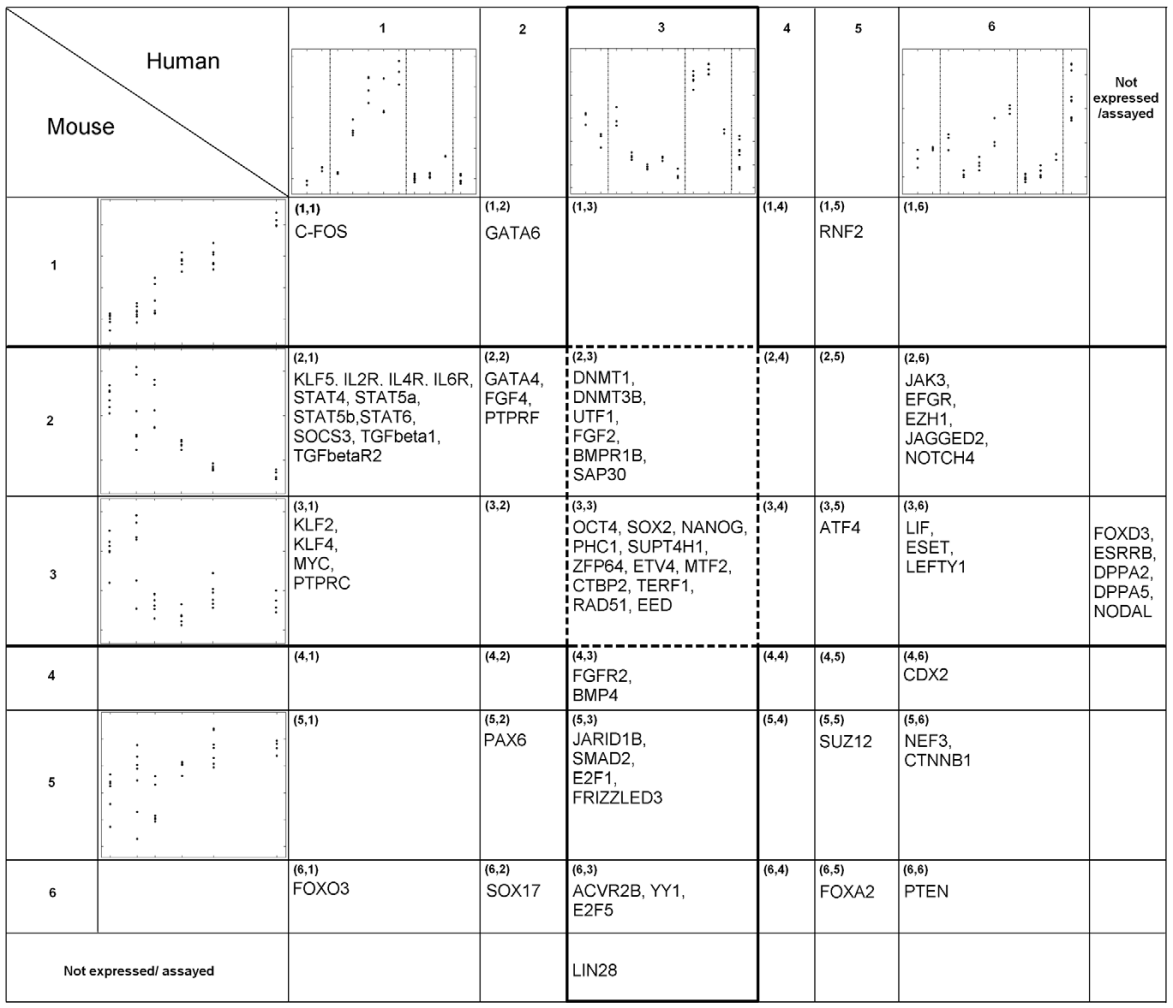
SCSC clusters of mES and hES cell differentiation data. Representative transcription regulators are listed in each cluster. Thick lines enclose clusters with upregulation in either mouse or human ES cells. Dotted lines enclose the conserved clusters with upregulation in ES cells of both species. Detailed expression patterns of every cluster and sample information are given in Figure S2.

We generated detailed time-course microarray data during a differentiation process of mES cells (GEO accession number: GSE12550, see Methods). Using SCSC, we jointly analyzed them with four datasets of undifferentiated and differentiated hES cells. The mES cell data were generated at eight time points during differentiation, with an average of six biological replicates at each time point (see Methods). The four human datasets included undifferentiated and differentiated cells from multiple American and European ES cell lines [30],[31],[32] together with two differentiation pathways of adult stem cells [33] (Figure S2A). We ran SCSC on 6,088 pairs of probe sets, representing an unbiased selection of orthologous genes that may best reflect the gene regulatory networks in mES and hES cells (Text S2). SCSC generated a result of 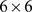 clusters (Figure 2).

### Overview of clustering results

Clusters (2, *)FF and (3, *) were upregulated in mES cells, and Cluster (*, 3) was upregulated in hES cells (Figure 2). Here * denotes all the indices from 1 to 6. For example, (*, 3) includes the clusters (1, 3), (2, 3) … (6, 3). The other mouse and human clusters had increasing expression patterns during differentiation. Clusters (2, 3) and (3, 3) had conserved upregulation in mES and hES cells. The part of the gene regulatory circuit that is conserved between mES and hES cells was represented in these two clusters (Table S3A). The gene pairs that belonged to Clusters (2, *) and (3, *) but did not belong to Clusters (2, 3) and (3, 3) were specifically expressed in mES cells. These genes represent the part of the gene regulatory network that are expressed in mES cells, but is disrupted in hES cells (Table S3B). Finally, gene pairs belonging to (*, 3) but not Clusters (2, 3) and (3, 3) were specifically expressed in hES cells (Table S3C).

**Figure 3:**
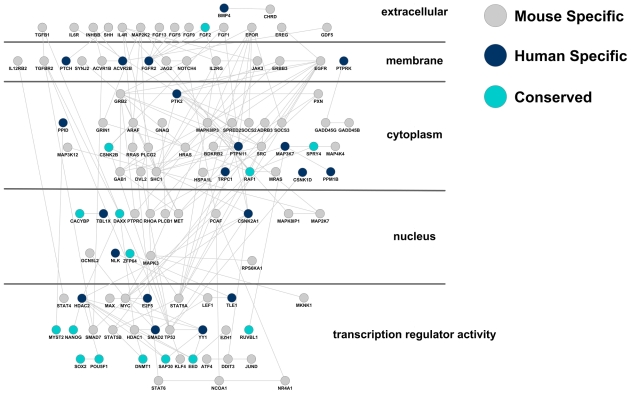
Induced components of signaling pathways in hES and mES cells. The gene induced in either hES or mES cells, i.e., Clusters (2, *), (3, *) and (*, 3), are mapped onto all the signaling pathways documented in the KEGG database [33] and plotted using Cytoscape software (www.cytoscape.org). Gray, blue and green nodes represent genes that are induced in hES cells only, mES cells only or both (conserved), respectively. The edges between any two nodes represent known protein-protein interactions documented in Cytoscape.

To explore which signaling pathways and what components of these signaling pathways are induced in hES and mES cells, we mapped the genes that were induced in either hES or mES cells, i.e., Clusters (2, *), (3, *) and (*, 3), onto all known signaling pathways documented in the KEGG pathway database [34]. The ES-induced components of these signaling pathways were plotted to highlight the hES-specific, mES-specific, and the conserved components (Figure 3).

### Conserved regulatory mechanisms in ES cells

Among 1,113 genes involved in transcriptional regulation (GO: 0003700) and included in this analysis, 448 clustered in either mES or hES upregulated clusters ((2, *), (3, *) and (*, 3)), indicating that a very large proportion (40%) of the transcriptional regulators were utilized in ES cells. Among these 448 transcription regulators, 85 (19%) exhibited conserved upregulation in mES and hES cells (in clusters (2, 3) and (3, 3)), representing a core set of regulators with higher expression in undifferentiated than differentiated ES cells (Table S4). Among these regulators, OCT4 and SOX2 are indispensable for maintaining an ES cell phenotype and for reprogramming [28]; NANOG, UTF1, and polycomb group proteins EED and PHC1 either promote self-renewal or inhibit differentiation. Repression of lineage-specific differentiation genes is critical for maintaining the undifferentiated state [35]. Conserved transcriptional repressors and corepressors included DNA methylation enzymes DNMT1 and DNMT3B, Polycomb group factors EED and PHC1, histone deacetylase SAP30, and transcription factors SUPT4H1, E2F8, TGIF1 and CTBP2. In addition, certain aspects of DNA replication and cell cycle regulation were also conserved in ES cells, as exemplified by conserved expression of CDK2, RAD51, E2F8, MYST2, POLYA1 and TERF1.

### The KLF regulatory module is required for pluripotency only in mES cells

**Figure 4:**
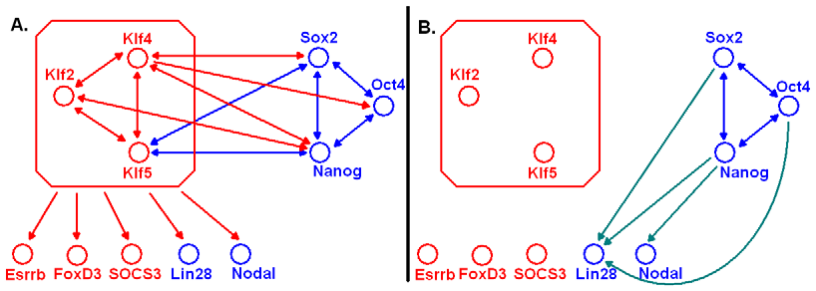
Rewiring of the KLF regulatory module. Nodes represent upregulated genes in ES cells in a conserved (blue, upregulated in both hES and mES cells) or species-specific (red, upregulated in mES cells only) manner. Edges represent positive regulatory relationships (activation) that are validated by ChIP-chip and RNAi data in both species (dark blue), in mouse only (red), or in human only (light blue). As the KLF module appears to have lost its regulatory function in hES cells, its target genes *ESRRB*, *FOXD3* and *SOCS3* have consistently lost their upregulation in hES cells as well (A). However, *LIN28* and *NODAL*, which are upregulated by the KLF module in mES cells, remain upregulated in hES cells. Their upregulation in hES cells might be activated by NANOG and OCT4 instead (B).

KLF2, KLF4 and KLF5 belong to the Krüppel-like factor (KLF) family of evolutionarily conserved zinc finger transcription factors that regulate numerous biological processes, including proliferation, differentiation, development and apoptosis [36]. We previously demonstrated that simultaneous depletion of KLF2, KLF4 and KLF5 led to differentiation of mES cells [37]. Consistent with this result, in mES cells, KLF2, KLF4 and KLF5 co-clustered with other pluripotency regulators (Clusters (2,*) and (3,*)), including OCT4, SOX2 and NANOG. Chromatin immunoprecipitation coupled to microarray assay (ChIP-chip) data showed that KLF2, KLF4 and KLF5 proteins all bind upstream of their own coding genes as well as upstream of *OCT4*, *SOX2* and *NANOG* [37]. NANOG and SOX2 ChIP-chip data demonstrated that they both bind to *KLF2*, *KLF4* and *KLF5* [38]. The co-clustering result together with the published RNA knockdown data and ChIP-chip data suggest that *KLF2*, *KLF4* and *KLF5* form a regulatory module that is coupled with the *OCT4-SOX2-NANOG* regulatory module in mES cells (Figure 4A).

The mES cell expression of the three KLF factors was not conserved in humans. Human *KLF2*, *KLF4* and *KLF5* were clustered in Cluster (*, 1), which exhibited low expression in hES cells and a steady increase during spontaneous and lineage-specific differentiation. This led to the hypothesis that the KLF2/4/5 module was decoupled from the OCT4-SOX2-NANOG module in the transcription network of hES cells. To explore the decoupling hypothesis, we first re-examined the mouse data for potential clues. In mES cells, the KLF2/4/5 regulatory module and the OCT4-SOX2-NANOG regulatory module were firmly established, because every factor bound to every other gene within the module. A maximum of 30 regulator-target links among the six transcription factors were allowed (Figure 4A). All except four of the allowed regulator-target links were confirmed by ChIP-chip data. The four missing links were OCT4->*KLF2/4/5* and SOX2->*KLF2*. All of these missing edges were between the two modules, which seemed to poise them for decoupling. Second, we checked if the inter-module regulatory links were preserved in hES cells. Human ChIP-chip data [39] showed that three out of the five inter-module regulatory links were dissociated (SOX2->*KLF4*, NANOG->*KLF4*, NANOG->*KLF2*). The two observations above and co-expression result were consistent with the hypothesis that the two regulatory modules were decoupled in hES cells.

If the KLF2/4/5 module was mouse-specific, it should *specifically* regulate other regulatory factors in mES cells. Therefore, the existence of species-specific targets of KLF2/4/5 could be further evidence for the decoupling hypothesis. Besides the three KLF genes themselves, *ESRRB*, *FOXD3* and *SOCS3* were among their specific targets in mES cells. ESRRB [40], FOXD3 [25] and SOCS3 [41] were all related to self-renewal and inhibiting differentiation in mES cells.FFFF In mice, *KLF2*, *KLF4*, and *KLF5* and *ESRRB*, *FOXD3*, and *SOCS3* all exhibited high expression in undifferentiated ES cells, and their expression decreases during differentiation. Moreover, *ESRRB*, *FOXD3* and *SOCS3* upstream regions were bound by KLF2, KLF4 and KLF5 in mES cells [37]. In humans, the expression levels of *ESRRB* and *FOXD3* dropped below a detectable level in all measured ES cells. Human *KLF2*, *KLF4*, *KLF5* and *SOCS3* were clustered in Cluster (1, *), implying that their expression increases as hES cells differentiate. In summary, with the decoupling of the KLF module from the OCT4-SOX2-NANOG module in hES cells, the upregulation of ESRRB, FOXD3 and SOCS3 in undifferentiated hES cells was diminished (Figure 4B).

Another group of KLF target genes in mice exhibited conserved upregulation in hES cells. ChIP-chip and RNAi data [37] confirmed that this group included *OCT4*, *SOX2*, *NANOG*, *LIN28* and *NODAL* (Figure 4A). In particular, *LIN28* and *NODAL* were upregulated by KLFs in mES cells, because KLFs bound to these genes in vivo and knocking-down KLFs substantially decreased their expression levels. Since *KLF2*, *KLF4* and *KLF5* themselves were not upregulated in hES cells, the maintenance of upregulation of *LIN28* and *NODAL* in hES cells may require *rewiring* of the transcription networks [23]. In other words, the upregulation of *LIN28* and *NODAL* in hES cells had to be achieved by transcription factors other than the KLFs. Consistent with this hypothesis, ChIP-chip data [37],[39] showed that OCT4, SOX2 and NANOG bound to *LIN28* in hES cells but not in mES cells; NANOG bound to *NODAL* in hES but not in mES cells (Figure 4B). As controls, none of *ESRRB*, *FOXD3* or *SOCS3* upstream was bound by OCT4, SOX2 or NANOG in hES cells.

In summary, the mouse-specific KLF2/4/5 regulatory module upregulated a set of key mES cell regulators. This module was not conserved in humans and therefore represented a peripheral component of the pluripotency maintaining regulatory networks. *KLF4* was included in the set of genes for reprogramming both mouse [42] and human cells [28]; However, *KLF4* was dispensable for maintaining the ES cell phenotype [28],[42]. This fact supports our hypothesis that genes involved in peripheral components of ES cell transcription networks should be capable of assisting but may not be essential for reprogramming.

### Empirical evaluation of SCSC results with independent experimental data

To what extent do gene clusters reflect functionally related gene groups? Although we do not expect a generic answer to this question, well-deliberated quantitative analyses may provide useful empirical data. Two sets of co-regulated genes were derived from an independent functional analysis, where seven regulatory proteins were knocked down by RNAi in mES cells [40]. To evaluate the consistency between the clustering result and the independently identified co-regulated genes, we applied a recently developed metric called the biological homogeneity index (BHI) [43]. BHI is the average proportion of gene pairs that are consistently allocated to both the same cluster and the same functional group in the gold standard dataset. A greater BHI reflects a higher consistency between the clusters and the functional groups. Because the gold standard datasets were generated from mES cells, we compared SCSC results with K-means clustering and hierarchical clustering performed on the same genes in the mES cell differentiation dataset (Figure S3). For a fair comparison, the same number of clusters were generated from K-means and hierarchical clustering as from SCSC. We gave K-means the advantage of starting from multiple initial values, minimizing the chances of being trapped by local maxima. In both comparisons, SCSC generated far more consistent gene groups with the functional groups defined by the independent RNAi studies, supporting the original intuition behind SCSC, that functional gene groups could be better revealed by comparative transcriptome analysis.

## Discussion

The applications of clustering analyses of expression data are limited by strong noise in the results. Some genes known to be involved in a particular pathway are invariably missed, whereas other apparently unrelated genes exhibit expression profiles that are strikingly similar to bona fide pathway components [21]. These shortcomings are explained in part by the observation that many microarray studies failed to sufficiently sample the biological variability within a system [21]. In light of this argument, transcriptomes of several organisms undergoing a similar biological process might be analyzed as one system with evolutionary distance providing the biological variability. Consequently, statistical development of cross-species clustering algorithms should enable assessment of expression conservation and diversity across species, thereby generating more functionally coherent gene groups. SCSC was built under this premise (Text S4).

### Other non-essential reprogramming factors

Similar to KLF4, *LIN28*, a transcriptional target of the KLF2/4/5 regulatory module [37], was also used as a reprogramming factor, but it was non-essential [29]. MYC, a transcription factor down-regulated during mES cell differentiation but not during hES cell differentiation (Figure 2), was another non-essential reprogramming factor [28],[42]. These examples highlight the power of cross-species analysis to distinguish core versus peripheral components of a transcription network for maintaining a particular phenotype.

### Alternative implementations of signaling pathways in mES and hES cells

Compared to differentiated cells, relatively few signal transduction factors were produced in ES cells. Comparing within the clusters that were upregulated in either mES or hES cells, i.e., among Clusters (2, *), (3, *) and (*, 3), genes involved in NOTCH, WNT, TGFβ, JAK-STAT and MAPK pathways were all depleted in the conserved clusters ((2, 3) and (3, 3), p-value <4*10^−5^). The lack of shared signal transduction factors in the conserved clusters suggests that these signaling pathways either do not present in one of the two ES cells or they utilize alternative implementations in them (Table 1).

**Table 1:** Distribution of genes participating in six signaling pathways in the ES clusters.

Pathway	Components	Mouse specific: Clusters (2, *), (3, *) but not (2, 3), (3, 3)	Human specific: Clusters (*, 3), but not (2, 3), (3, 3)	Shared: Clusters (2, 3), (3, 3)	Comment
JAK-STAT	Extracellular & membrane	LIF, IL2R, IL4R, IL6R			mES specific
	Downstream factors	JAK3, TK2^#^, PTPRC, PTPRF, PTPRN, SOSC3, PINK1*			
	Transcription regulators	STAT3^#^, STAT4, STAT5A, STAT5B, STAT6			
NOTCH	Extracellular & membrane	NOTCH4, JAGGED2, MFNG			
	Downstream factors				
	Transcription regulatorsF	NCOA1*			
TGFβ	Extracellular & membrane	TGFβ1, TGFβR1	BMP4, ACVR2B	LEFTY, BMPR1	Alternatively implemented
	Downstream factors	MAPK3, PINK1*	PPP1CC*, SAR1A		
	Transcription regulators	[SMAD7]^&^, SKIL^&^, NCOA1*	[SMAD2]^&^ (interact with TGIF1)	TGIF1^&^	
WNT	Extracellular & membrane	[FRIZZLED9], H{LRP5}	[FRIZZLED3], {LRP6}	[FRIZZLED7]	
	Downstream factors	RHOA	CSNK2A1, CSNK1D	CSNK2B	
	Transcription regulators	[TLE4]^&^, LEF1, MYC	[TLE1]^&^, TCF7L2	CTBP2^&^	
MAPK	Extracellular & membrane	FGF4, MET*, EGFR, GRB2	FGFR2	{FGF2}, TDGF1	
	Downstream factors	[ARAF], {PTPRC}, {PTPRF}, {PTPRN}, [MAP3K6], [MAP2K7], [MAPK3]	[KRAS], {PTPRK}, {PRPRG}, {PTPN11}, [MAP3K7]	[RAF1]	
	Transcription regulators	ATF4			
VEGF	Extracellular & membrane				Unlikely to be expressed in ES cells
	Downstream factors	PINK1*, PLCD1, PLA2	PTK2, PPP1CC*, CLK2		
	Transcription regulators				

Genes in the same family are embraced with the same parenthesis. Genes with a * are involved in the multiple pathways. Genes with ^&^ signs are transcriptional repressors or co-repressors. Genes with a ^#^ have abundant transcripts in mES cells, but they do not show obvious up or down regulation during differentiation of mES cells.

JAK-STAT and NOTCH were present in mES cells, but no typical signaling transducers of these pathways appeared to be present in hES cells. It has long been known that mES cells remain undifferentiated in the presence of Leukemia Inhibitory Factor (LIF), and the activation of Signal Transducer and Activator of Transcription 3 (STAT3) via LIF-JAK signaling appears sufficient for maintenance of pluripotency of mES cells. However, LIF is unable to maintain the pluripotent state of hES cells [44]. The mechanism behind this apparent discrepancy is not fully understood, although the activation of human STAT3 alone does not sustain self-renewal of hES cells [44]. As summarized in Table 1, none of the key components of the JAK-STAT pathway active in mES cells were present in hES cells, including key kinases JAK3 and TK2 and a family of five STAT transcription factors. This indicates that the JAK-STAT pathway is poised to receive the LIF signal in mES cells. Although only STAT3 is known as a downstream factor of LIF signaling in mES cells, our data predict that the other four STAT transcription factors may also contribute to maintaining the mES cell phenotype, since all of these genes are downregulated during differentiation.

TGFβ, WNT and MAPK pathways appeared to be present in both mES and hES cells. However, our data suggest that mouse and human ES cells do not always use orthologous factors in these pathways. The non-orthologous components of these signaling pathways appeared to share two common features. First, paralogous members of the same gene family could serve as surrogates of an orthologous component. Using the WNT pathway as an example, growth factors FRIZZLED9 (mES), FRIZZLED3 (hES), receptors LRP5 (mES) and LRP6 (hES), and transcription regulators HHTLE4HH (mES) and TLE1 (hES) were alternative members of the same gene family that appeared to assume orthologous functions in mES and hES cells (Table 1). Second, the alternatively implemented signaling transduction routes in the two species appeared to share the same regulatory logic. For example, TGFβ signaling in mES cell is inhibited by SMAD7 [45] and SKILl at the receptor and transcriptional levels [46], whereas in the hES cell, another inhibition mechanism appeared to be present through the interaction of SMAD2 and TGIF1 [47]. Also, WNT signals to HHTLE4HH and TLE1 in mES and hES cells, respectively, for probably the same purpose of transcriptional repression [48]. The characterization of species-specific signaling pathways and alternative routes of signaling transduction facilitates understanding how pluripotency is maintained in mES and hES cells and why a signal could induce seemingly different and even reverse responses from these cells (BMP: [49],[50], WNT: [51],[52]; LIF: [44]).

### Mouse epiblast stem cells

mES and hES cells are similar in the sense that they are both derived from the inner cell mass of blastocyst embryos, and are both pluripotent. Besides mES cells, pluripotent stem cells were also derived from the late epiblast layer of post-implantation mouse embryos (mEpiS cells) [53]. Compared to mES cells, mEpiS cells are functionally more similar to hES cells in the following ways. Both hES and mEpiS cells, but not mES cells, can differentiate into trophoblast upon exposure to Bmp4; display very limited capacity for chimera formation when injected or aggregated with mouse preimplantation embryos; form relatively large and flat colonies when grown as monolayers; do not survive well as individual cells. Importantly, the pluripotency of hES cells and mEpiS cells, can be maintained via Activin/Nodal signaling [53],[54], whereas Activin induces mES cells to differentiate into mesendoderm [55]. Thus, the alternative implementations of gene regulatory networks between hES can mES cells may reflect their functional differences and indicate the differences of their seemingly comparable temporal origins during embryonic development.

## Methods

### Transcription profiling of mouse ES cells

Total RNA for transcriptional profiling was obtained from B6 mES cells at various stages of differentiation, including undifferentiated (0 day), 4, 8, 12, 21 and 31 days of differentiation. Six biological samples were analyzed at each time point. B6 mouse ESC were cultured on mouse embryonic feeders (MEFs) using standard methods as previously described [56] in 15% FCS supplemented with LIF. Undifferentiated ES cell samples were obtained by trypsinising near confluent plates of ES cells and depleting the MEFs by plating the cells onto gelatin coated plates for 2×20 min. The ES on gelatin samples were MEF depleted ES cells seeded on gelatin coated dishes and cultured until they reached ∼70% confluency. To ensure the undifferentiated ES cell samples were free from MEF contamination, MEF depleted ES cells that passaged once on gelatin were used as 0-day ES cell samples. To make EBs, the ES cells on gelatin were seeded into non-adherent petri dishes, and LIF was withdrawn to induce differentiation. Half of the EB media was changed every 3–4 days. The formation of EBs was consistent with previous studies [57],[58]. After 8 days, numerous cystic structures were observed and became progressively larger over time. After about 10 days, beating foci of cardiac myocytes could be observed in some EBs, indicating the terminal differentiation of some cell types.

Total RNA was extracted from the different samples using the RNeasy kit (Quiagen) and amplified using a two-round linear amplification strategy as previously described [56]. The labeled RNA was then hybridized to Affymetrix MgU74Av2 microarrays according to the manufacturer's instructions. Normalization and probe-level modeling were done with dChip software [59].

### Statistical model

The expression value of an orthologous gene pair is denoted as (g_i_, g_i′_), where i and i′ index two orthologous genes. The goal of SCSC is to assign a cluster label c_i,i′_ to every orthologous gene pair (i, i′). The range of c_i,i′_ goes from (1, 1) to (K, L), where K and L are the maximum numbers of clusters allowed in the two species. Without loss of generalizability, we assume there are no more than K clusters in either of the two species; i.e.,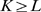, and then (K, K) are the largest possible values c_i,i′_ can take. The following statistical model does not assume K = L. However K = L is used in the SCSC program implementation.

SCSC takes a model-based approach. The cluster labels are assumed to be generated according to probabilities 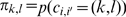 and that conform to a multinomial logit model [60]:
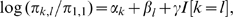
(1)where 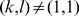. 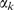
and 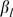 capture the independent co-expression information contributed by each species, i.e., row and column effects in Figure 2. 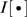 is an 0–1 indicator function. 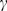 represents the degree of dependence between correspondent clusters between the two species. When 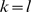, cluster 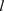 is deemed as the correspondent cluster to cluster k. The order of the result clusters in a clustering analysis is usually arbitrarily determined. SCSC orders its clusters in the two species in a way that the clusters in the two species with the largest intersection of orthologous genes are given the same numerical indicator (See below).

Given the cluster indicator of a gene pair, for example 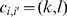, the model for complete data is: 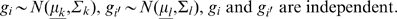
(2)Here 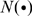 denotes a Gaussian distribution; 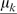
and 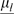 are the mean vectors of the *k* ^th^ and the 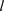 ^th^ clusters in the two species, respectively; 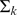 and 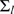 are their corresponding covariance matrices. A generative probabilistic model for two species gene expression data is:
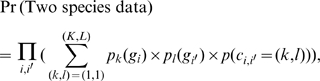
(3)where the product 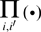 enumerates all gene pairs (i, i′); 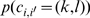 is the probability of gene pair (i, i′) coming from cluster 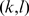; 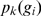 is the likelihood of gene i given it comes from cluster *k* in one species, and 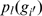 is the likelihood of gene i′ given it comes from cluster 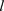 of the other species. An iterative maximization algorithm was developed to fit the SCSC model (Figure S4, Text S3). Because SCSC uses a likelihood maximization approach based on the EM algorithm, the local maximum issue that is general to EM algorithm applies. The SCSC program is available both as a downloadable program and as a web application at: http://biocomp.bioen.uiuc.edu/SCSC.

## Supporting Information

Text S1 Synthetic data.
(0.01 MB PDF)

Text S2 Selecting genes for SCSC analysis of mouse and human ES cells.
(0.01 MB PDF)

Text S3 An iterative maximization algorithm for SCSC model.
(0.03 MB PDF)

Text S4 SCSC algorithm.
(0.02 MB PDF)

Figure S1 Performance evaluation on synthetic datasets. Average performance scores from 20 independent runs of each algorithm are listed. Dataset numbers correspond to the datasets listed in Table S1. K-means, hierarchical clustering, MCLUST, WGNCA and CLICK were first executed on each species and then their results were summarized across the two species. The Random clustering is generated by choosing random centroids and assigning each data point into the nearest centroid. Three performance scores were used for comparison, average global scatter, average center scatter, and proportion of genes being assigned to wrong clusters. The definitions of the first two performance scores are as follows: 1. Average global scatter: the average distance between every data point and the cluster center that it was assigned to. For two species, the average global scatter was the sum of distances between every gene and the center of its own species divided by the total number of genes in two species. 2. Average center scatter: the average distance between true cluster centers and their corresponding computed cluster centers. In a perfect clustering result, center scatter equals 0. Center scatter for two species was computed by dividing the sum of center scatters in both species by the total number of clusters in both species.
(0.06 MB PDF)

Figure S2 SCSC clusters of mouse and human ES cell differentiation. (A) Sample information for human ES cells. (B) The number of orthologous probe sets in each result cluster, and (C) the corresponding expression patterns of mouse and human clusters. Each dot represents the mean expression of a cluster in a biological replicate.
(0.04 MB PDF)

Figure S3 Performance evaluation with independent experimental data. The consistency of a clustering result to a set of co-regulated gene groups is measured by biological homogeneity index (BHI). K-means clustering was run 10 times with different initial values. The red bars and their error bars represent the average BHI and the standard deviation for these 10 runs. The best performance out of the 10 runs is also reported (yellow bar). Two test sets of co-regulated genes groups were defined as follows. Set 1: Co-regulated genes were defined as the genes whose expression levels were affected by all of the seven RNA knockdown (RNAi) experiments of seven regulatory proteins (OCT4, SOX2, NANOG, ESRRB, TBX3, TCL1, DPPA4) which maintain ES cell identity. Each RNAi experiment provided a list of genes whose expression was affected. Taking the intersection of the seven gene lists, a total of 60 genes were identified as regulated by all seven ES cell regulators. These 60 genes were used as the first test set. Set 2: Co-regulated genes were defined as the genes whose expression was affected by RNAi knockdown of all four of the transcription factors NANOG, OCT4, SOX2 and ESRRB, and for which the direction of expression change was the same. These four transcription factors physically interact and synergistically regulate gene expression in ES cells. Two groups of co-regulated genes were identified. Group 1 contained 107 genes that were consistently induced by the RNAi of each of the four factors, whereas group 2 contained 48 genes that were repressed by all four RNAi treatments. These two co-regulated gene groups constitute the second test set.
(0.03 MB PDF)

Figure S4 Scheme of computational implementation of the SCSC method. The scheme mimics an EM algorithm for clustering one-species data under a Gaussian-mixture model. (Text S4)
(0.02 MB PDF)

Table S1 Synthetic datasets. Cluster number is the number of clusters in the two species. For example, means 10 clusters in both species. Dimension is the number of samples in each species. “# of data points in each cluster” is the number of orthologous genes in each cluster”. “# of scatter data points” is the number of randomly distributed gene pairs that do not belong to any clusters. They represent intrinsic deviation of the transcriptome from a clustering model. The cluster means of dataset 1–5 are randomly generated between 0 and 10. The cluster mean of dataset 6 are generated between 0 and 13. Cluster variation shows the standard deviation used to generate each cluster, with the two numbers representing two standard deviations for each of the two species.
(0.02 MB PDF)

Table S2 Performance evaluation with errors in ortholog map. 10%–30% of the ortholog mapping in synthetic dataset 1 (Table S1) are randomly permutated to represent the scenarios of errors in ortholog map. SCSC, K-means and DCA were executed on these perturbed datasets, and performance metrics were recorded.
(0.01 MB PDF)

Table S3 SCSC clusters. (A) with conserved upregulation in hES and mES cells, (B) specifically upregulated in mES cells, and (C) specifically upregulated in hES cells.
(0.15 MB PDF)

Table S4 Conserved transcription regulators in human and mouse ES cells. Genes with a & may act as transcriptional repressors or corepressors.
(0.01 MB PDF)
