# Supplementary material for: Modeling Co-Expression across Species for Complex Traits: Insights to the Difference of Human and Mouse Embryonic Stem Cells
Source: PLoS Comput Biol. 2010 Mar 12;6(3):e1000707. doi: 10.1371/journal.pcbi.1000707 (PMC2837392; doi:10.1371/journal.pcbi.1000707)
Supplement: Text S1 — Synthetic data. (0.01 MB PDF) [file pcbi.1000707.s001.pdf]

Text S1.

Synthetic data. Six synthetic datasets were generated (Table S1). In each of the first five synthetic dataset, 2275 to 4250 pairs of expression vectors were simulated, representing expression data from two species. The length of an expression vector ranged from 10 to 20, representing 10 to 20 microarray samples in each species. The number of clusters in each species ranged from 10 to 20. For each cluster, a mean vector was generated from a uniform distribution between 0 and 10, representing the range of log intensities of gene expression measurements. The expression of every gene in a cluster was simulated from a Gaussian distribution with the simulated mean and a given variance. Every cluster in one species had a correspondent cluster in the other species. Except for “scatter genes” (see below), all orthologous gene pairs were generated from correspondent clusters. It should be noted that the mean patterns of a pair of correspondent clusters were generated independently. Fifty to 275 scattered genes whose expression levels were randomly generated from a uniform distribution in each species were added to represent genes under random drift. The range of the uniform distribution for scatter genes was chosen from the range of all expression values that had been simulated so far. In dataset 6, 1280 gene pairs were simulated for two species. In each species, these 1280 genes were generated from four clusters. Each cluster had a given mean in the range of 0 to 13, representing the range of log intensities of Gene Expression Index [1] of Affymetrix GeneChip arrays. The mean values of a cluster change gradually across samples, mimicking a time-course experimental setting. Another 500 scatter genes were added to each species.

The performance of SCSC was compared with that of DCA, K-means, hierarchical clustering, MCLUST, MGCNA and CLICK. K-means, hierarchical clustering, MCLUST, MGCNA and CLICK were not designed for cross-species analysis. We therefore executed these clustering algorithms in each of the two species, and then for each cluster a conserved cluster in the other species was identified by whichever group had the largest overlap of orthologous genes. Bayesian Information Criterion (BIC) was used for SCSC to choose a cluster number. Because other methods were assigned manually with the

correct number of clusters. These artificial assignments disadvantaged SCSC in this comparison. For hierarchical clustering, we cut the result dendrogram at a proper depth to generate the desired number of clusters. We ran each algorithm 20 times independently on every dataset. Performance scores from the 20 runs were averaged to obtain a more robust performance indicator (Figure S1).

Reference to supplementary documents

1. Li C, Wong WH (2001) Model-based analysis of oligonucleotide arrays: expression index computation and outlier detection. Proc Natl Acad Sci U S A 98: 31-36.
