# Supplementary material for: Modeling Co-Expression across Species for Complex Traits: Insights to the Difference of Human and Mouse Embryonic Stem Cells
Source: PLoS Comput Biol. 2010 Mar 12;6(3):e1000707. doi: 10.1371/journal.pcbi.1000707 (PMC2837392; doi:10.1371/journal.pcbi.1000707)
Supplement: Text S2 — Selecting genes for SCSC analysis of mouse and human ES cells. (0.01 MB PDF) [file pcbi.1000707.s002.pdf]

Text S2.

Selecting genes for SCSC analysis of mouse and human ES cells. We first took the non-redundant union of the following gene groups in mouse: 1) Transcription factors as annotated by GO term: transcription factor activity (GO: 0003700); 2) genes involved in any of the following signaling pathways: WNT, NOTCH, MAPK, JAK-STAT, VEGF and TGF. GenMAPP and KEGG databases were used to identify these genes (non-redundant union was taken); 3) cell surface proteins (GO term: cell surface, 0009986); 4) nuclear receptors (GO term: ligand-dependent nuclear receptor activity: 0004879); 5) genes that are bound by either of the following proteins or protein complexes in mouse ES cells: OCT4, SOX2, NANOG and polycomb group, as identified from ChIP-chip studies [5,6]; 6) genes that were previously found to have higher expression in mouse ES cells than in differentiated cells [1]. The non-redundant union of the gene groups above resulted in 4106 genes (5195 probe sets). Probesets on the Human U133A array that target orthologous genes of these mouse genes were identified using the map of orthologous probesets provided by Affymetrix (www.affymetrix.com). This orthologous map contained pairwise orthologous information.

The gene union obtained above was then subjected to two filters: 1) If a gene, in either mouse or human, has a maximum gene expression index (computed by dChip: www.dchip.org) smaller than 50, it was regarded as not expressed and its gene pair was dropped. 2) If a gene in both mouse and human exhibited a coefficient of variation (standard deviation over mean) smaller than 0.1, it was regarded as invariant in the ES cell differentiation process and discarded. After implementation of these criteria, 6088 orthologous pairs of probe sets were passed onto SCSC analysis.

Reference to supplementary documents

1. Zhou Q, Chipperfield H, Melton DA, Wong WH (2007) A gene regulatory network in mouse embryonic stem cells. Proceedings of the National Academy of Sciences 104: 16438.
