# Supplementary material for: Modeling Co-Expression across Species for Complex Traits: Insights to the Difference of Human and Mouse Embryonic Stem Cells
Source: PLoS Comput Biol. 2010 Mar 12;6(3):e1000707. doi: 10.1371/journal.pcbi.1000707 (PMC2837392; doi:10.1371/journal.pcbi.1000707)
Supplement: Text S3 — An iterative maximization algorithm for SCSC model. (0.03 MB PDF) [file pcbi.1000707.s003.pdf]

Text S3.

An iterative maximization algorithm for SCSC model. SCSC implements an iterative maximization algorithm that mimics the EM algorithm [1] for clustering one-species data under a Gaussian-mixture model [2] (Figure S4). This algorithm iterates among three steps. We name the three steps E-step, M-step and logistic regression step, respectively. In the E-step, a two-dimensional clustering indicator $c_{i,i'}$ is assigned as (k,l) for gene pair (i, i'), according to the following probability:

$$p(c_{i,i'}=(k,l)) = a \times p(g_i,\theta_k) \times p(g_{i'},\theta'_l) \times \hat{\pi}(k,l) \quad (4),$$

where a is a normalization constant; $\hat{\pi}(k,l)$ denotes the prior probability of $c_{i,i'}=(k,l)$ before seeing the expression data; $p(g_i,\theta_k)$ and $p(g_{i'},\theta'_l)$ denote the conditional probabilities of gene i and i', respectively, given their cluster indicators. In the M-step, parameters are updated to maximize $p(g_i,\theta_k)$ and $p(g_{i'},\theta'_l)$, given expression data and $c_{i,i'}$. It should be noticed that given $c_{i,i'}$, $p(g_i,\theta_k)$ and $p(g_{i'},\theta'_l)$ are independent, and therefore they can be maximized separately. Before the next step, we rearrange the order of the clusters in the two species, such that the first clusters the two species have the largest overlap among all pairs of clusters; excluding the first clusters in both species, the second clusters in the two species have the largest overlap in the remaining clusters; and so on; until all the clusters are paired or all the clusters in the species with a smaller cluster number are paired. In the logistic regression step, the numbers of gene pairs in the clusters are used to first estimate parameters α,β and γ. With the fitted α, β and γ, the re-estimated proportion of gene pairs in each cluster is given by:

$$\hat{\pi}_{k,l} = \exp(\hat{\alpha}_k + \hat{\beta}_l + \hat{\gamma} I[k=l]) \Big/ (1 + \sum_{(k,l)\neq(1,1)} \exp(\hat{\alpha}_k + \hat{\beta}_l + \hat{\gamma} I[k=l])) \quad (5)$$

for $(k,l) \neq (1,1)$, and

$$\hat{\pi}_{1,1} = 1 \Big/ (1 + \sum_{(k,l)\neq(1,1)} \exp(\hat{\alpha}_k + \hat{\beta}_l + \hat{\gamma} I[k=l])) \quad (6).$$

If there is non-trivial trend in the data for orthologous genes to co-appear in correspondent clusters, γ will be fitted as a positive value, and the re-estimated $\hat{\pi}_{k,l}$ will

be strengthened (becomes larger) for the correspondent clusters ($k = l$). Otherwise γ will be close to 0 and all $\hat{\pi}_{k,l} \approx \pi_{k,l}$. In the E-step of the next iteration, the probability of $c_{i,i'} = (k,l)$ is computed from the re-estimated proportions of gene pairs in each cluster $\hat{\pi}(k,l)$. Finally, if the result of the M-step is directly fed back into the next E-step, this algorithm degenerates into a Gaussian-mixture model for independently clustering data from each of the two species. We tested the algorithm on a variety of simulated and real datasets. For every dataset, the likelihood evaluated by equation (3) always increased during the iterations (data not shown).

Reference to supplementary documents

1. Dempster A, Laird N, Rubin D (1977) Maximum likelihood from incomplete data via the EM algorithm. J Roy Statist Soc B 39: 1-38.
2. Banfield J, Raftery, AE (1993) Model-based Gaussian and non-Gaussian clustering. Biometrics 49.: 803-821.
