# Supplementary material for: Modeling Co-Expression across Species for Complex Traits: Insights to the Difference of Human and Mouse Embryonic Stem Cells
Source: PLoS Comput Biol. 2010 Mar 12;6(3):e1000707. doi: 10.1371/journal.pcbi.1000707 (PMC2837392; doi:10.1371/journal.pcbi.1000707)
Supplement: Text S4 — SCSC algorithm. (0.02 MB PDF) [file pcbi.1000707.s004.pdf]

Text S4.

SCSC algorithm. SCSC mimics an EM algorithm for clustering one-species data under a Gaussian-mixture model (Figure S4). In the E-step, a two-dimensional clustering indicator $c_{i,i'}$ is assigned to (k,l) for gene pair (i, i'). $\hat{\pi}(k,l)$ is the probability of $c_{i,i'} = (k,l)$ without seeing the expression data. $p(g_i, \theta_k)$ and $p(g_{i'}, \theta_l')$ are the conditional probabilities of gene i and i', respectively, given their cluster indicators. In the M-step, parameters are updated according to expression data and $c_{i,i'}$. This was followed by a logistic regression step that re-estimates the number of gene pairs in the clusters. The re-estimated distribution of gene pairs in the clusters fit into the E-step in the next iteration.

Beyond SCSC model. Our results demonstrate that the logistic regression model in SCSC captures a simple correlation structure of the cluster memberships between the two species. More complicated correlation structures are plausible and can be explored, if such correlation is supported by data and relevant biological context. Moreover, SCSC does not assume any analytical relationship between the average expression patterns of the correspondent clusters. This avoids the need for matching the cell and environmental conditions across species, which is a major difficulty in direct comparison of gene expression values of orthologous genes [1]. However, multi-species data generated from well controlled experimental conditions could enable explicit exploration of the correlation of the average expression patterns across species [2]. On top of such data, modeling the functional relationship between the average expression patterns across correspondent clusters may increase the power of borrowing the strengths of data across-species. Beyond the gene expression domain, novel methods have also been developed for co-evolution [3] and conservation [4] analyses of interacting proteins. Although protein-protein interaction is only partially reflected by co-expression, it would be interesting to correlate protein interaction and co-expression analyses in future research.

Reference to supplementary documents

1. Tirosh I, Bilu Y, Barkai N (2007) Comparative biology: beyond sequence analysis. Curr Opin Biotechnol 18: 371-377.
2. Wang S, He F, Xiong W, Gu S, Liu H, et al. (2007) Polycomblike-2-deficient mice exhibit normal left-right asymmetry. Dev Dyn 236: 853-861.
3. Fraser HB, Hirsh AE, Wall DP, Eisen MB (2004) Coevolution of gene expression among interacting proteins. Proc Natl Acad Sci U S A 101: 9033-9038.
4. Karimpour-Fard A, Detweiler CS, Erickson KD, Hunter L, Gill RT (2007) Cross-species cluster co-conservation: a new method for generating protein interaction networks. Genome Biol 8: R185.
