# Supplementary material for: Modeling Co-Expression across Species for Complex Traits: Insights to the Difference of Human and Mouse Embryonic Stem Cells
Source: PLoS Comput Biol. 2010 Mar 12;6(3):e1000707. doi: 10.1371/journal.pcbi.1000707 (PMC2837392; doi:10.1371/journal.pcbi.1000707)
Supplement: Figure S1 — Performance evaluation on synthetic datasets. Average performance scores from 20 independent runs of each algorithm are listed. Dataset numbers correspond to the datasets listed in Table S1. K-means, hierarchical clustering, MCLUST, WGNCA and CLICK were first executed on each species and then their results were summarized across the two species. The Random clustering is generated by choosing random centroids and assigning each data point into the nearest centroid. Three performance scores were used for comparison, average global scatter, average center scatter, and proportion of genes being assigned to wrong clusters. The definitions of the first two performance scores are as follows: 1. Average global scatter: the average distance between every data point and the cluster center that it was assigned to. For two species, the average global scatter was the sum of distances between every gene and the center of its own species divided by the total number of genes in two species. 2. Average center scatter: the average distance between true cluster centers and their corresponding computed cluster centers. In a perfect clustering result, center scatter equals 0. Center scatter for two species was computed by dividing the sum of center scatters in both species by the total number of clusters in both species. (0.06 MB PDF) [file pcbi.1000707.s005.pdf]

Figure S1: Performance evaluation on synthetic datasets. Average performance scores from 20 independent runs of each algorithm are listed. Dataset numbers correspond to the datasets listed in Table S1. K-means, hierarchical clustering, MCLUST, WGNCA and CLICK were first executed on each species and then their results were summarized across the two species. The Random clustering is generated by choosing random centroids and assigning each data point into the nearest centroid. Three performance scores were used for comparison, *average global scatter*, *average center scatter*, and proportion of genes being assigned to wrong clusters. The definitions of the first two performance scores are as follows:

1. *Average global scatter*: the average distance between every data point and the cluster center that it was assigned to. For two species, the average global scatter was the sum of distances between every gene and the center of its own species divided by the total number of genes in two species.
2. *Average center scatter*: the average distance between true cluster centers and their corresponding computed cluster centers. In a perfect clustering result, center scatter equals 0. Center scatter for two species was computed by dividing the sum of center scatters in both species by the total number of clusters in both species.

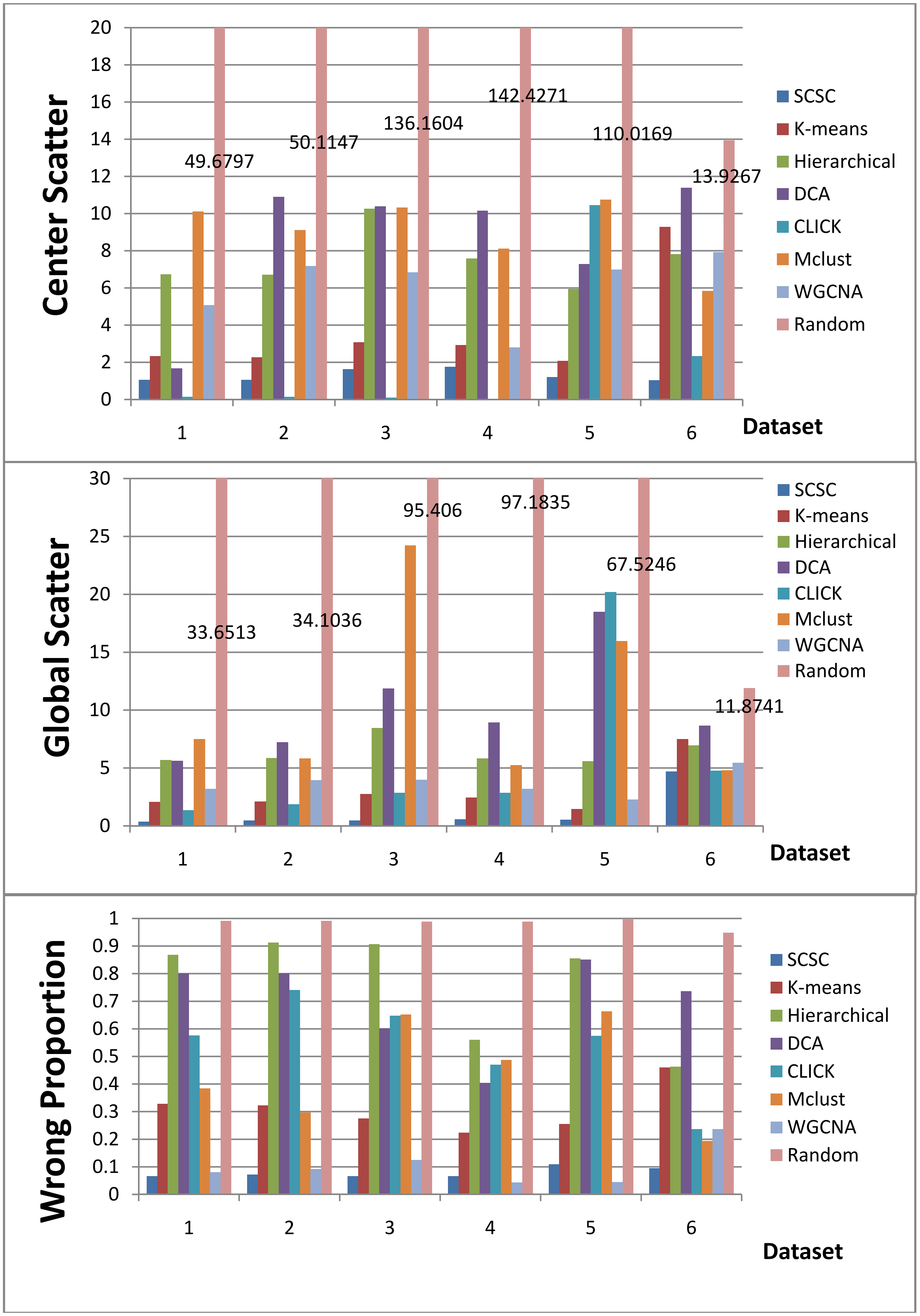

Center Scatter
20
18
16
14
12
10
8
6
4
2
0
49.6797
50.1147
136.1604
142.4271
110.0169
13.9267
1
2
3
4
5
6
Dataset
SCSC
K-means
Hierarchical
DCA
CLICK
Mclust
WGCNA
Random
Global Scatter
30
25
20
15
10
5
0
33.6513
34.1036
95.406
97.1835
67.5246
11.8741
1
2
3
4
5
6
Dataset
SCSC
K-means
Hierarchical
DCA
CLICK
Mclust
WGCNA
Random
Wrong Proportion
1
0.9
0.8
0.7
0.6
0.5
0.4
0.3
0.2
0.1
0
1
2
3
4
5
6
Dataset
SCSC
K-means
Hierarchical
DCA
CLICK
Mclust
WGCNA
Random
