# Supplementary material for: Modeling Co-Expression across Species for Complex Traits: Insights to the Difference of Human and Mouse Embryonic Stem Cells
Source: PLoS Comput Biol. 2010 Mar 12;6(3):e1000707. doi: 10.1371/journal.pcbi.1000707 (PMC2837392; doi:10.1371/journal.pcbi.1000707)
Supplement: Figure S2 — SCSC clusters of mouse and human ES cell differentiation. (A) Sample information for human ES cells. (B) The number of orthologous probe sets in each result cluster, and (C) the corresponding expression patterns of mouse and human clusters. Each dot represents the mean expression of a cluster in a biological replicate. (0.04 MB PDF) [file pcbi.1000707.s006.pdf]

Figure S2: SCSC clusters of mouse and human ES cell differentiation. (A) Sample information for human ES cells. (B) The number of orthologous probe sets in each result cluster, and (C) the corresponding expression patterns of mouse and human clusters. Each dot represents the mean expression of a cluster in a biological replicate.

A

Symbols	Cell lines	Reference
A1	Undifferentiated H1 ES cells	[1]
A2	Differentiated H1 ES cells	
B1	ES cells	[2]
B2	Hematopoietic stem/progenitor cells (HSPC)	
B3	Hematopoietic differentiated cells (HDC)	
B4	Epidermis' keratinocytes stem/progenitor cells (KSPC)	
B5	Epidermis differentiated cells, (KDC)	
C1	Finnish ES cells (FES)	[3]
C2	Swedish ES cells (HS)	
C3	Fibroblasts	
D	HSF-1, HSF-6 and H9 ES cells	[4]

B

Cluster index of mouse data \ Cluster index of human data	**1**	**2**	**3**	**4**	**5**	**6**
**1**	291	190	122	266	188	200
**2**	317	224	150	174	106	238
**3**	178	177	236	98	116	125
**4**	237	141	95	136	90	231
**5**	132	75	233	149	186	41
**6**	245	157	111	162	135	136

C

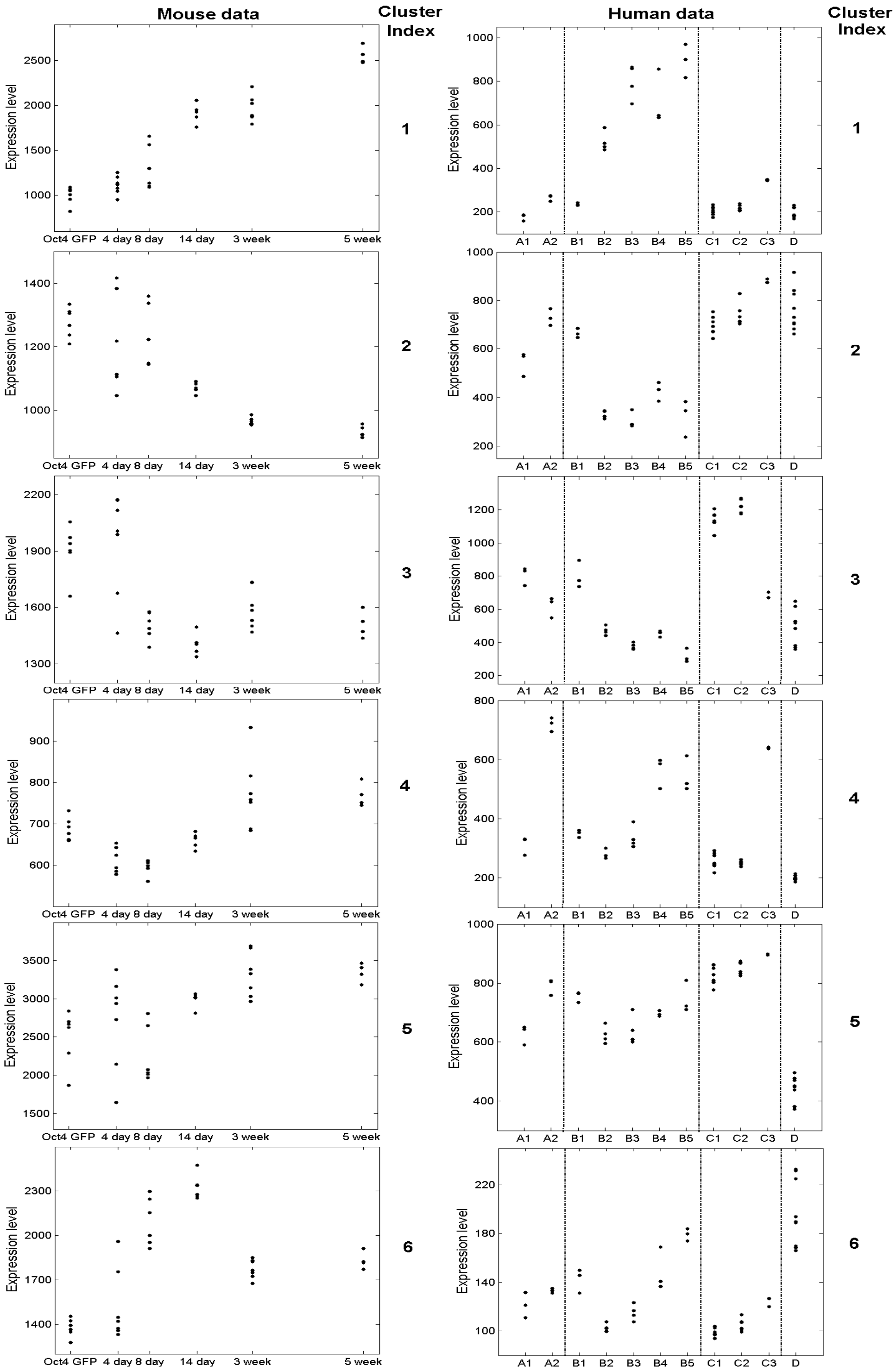
Mouse data
Cluster Index
Human data
Cluster Index
Expression level
Oct4 GFP
4 day
8 day
14 day
3 week
5 week
A1
A2
B1
B2
B3
B4
B5
C1
C2
C3
D
1
2
3
4
5
6
