# Supplementary material for: Modeling Co-Expression across Species for Complex Traits: Insights to the Difference of Human and Mouse Embryonic Stem Cells
Source: PLoS Comput Biol. 2010 Mar 12;6(3):e1000707. doi: 10.1371/journal.pcbi.1000707 (PMC2837392; doi:10.1371/journal.pcbi.1000707)

Figure S3. Performance evaluation with independent experimental data. The consistency of a clustering result to a set of co-regulated gene groups is measured by biological homogeneity index (BHI). K-means clustering was run 10 times with different initial values. The red bars and their error bars represent the average BHI and the standard deviation for these 10 runs. The best performance out of the 10 runs is also reported (yellow bar). Two test sets of co-regulated genes groups were defined as follows.

Set 1: Co-regulated genes were defined as the genes whose expression levels were affected by all of the seven RNA knockdown (RNAi) experiments of seven regulatory proteins (OCT4, SOX2, NANOG, ESRRB, TBX3, TCL1, DPPA4) which maintain ES cell identity. Each RNAi experiment provided a list of genes whose expression was affected. Taking the intersection of the seven gene lists, a total of 60 genes were identified as regulated by all seven ES cell regulators. These 60 genes were used as the first test set.

Set 2: Co-regulated genes were defined as the genes whose expression was affected by RNAi knockdown of all four of the transcription factors NANOG, OCT4, SOX2 and ESRRB, and for which the direction of expression change was the same. These four transcription factors physically interact and synergistically regulate gene expression in ES cells. Two groups of co-regulated genes were identified. Group 1 contained 107 genes that were consistently induced by the RNAi of each of the four factors, whereas group 2 contained 48 genes that were repressed by all four RNAi treatments. These two co-regulated gene groups constitute the second test set.

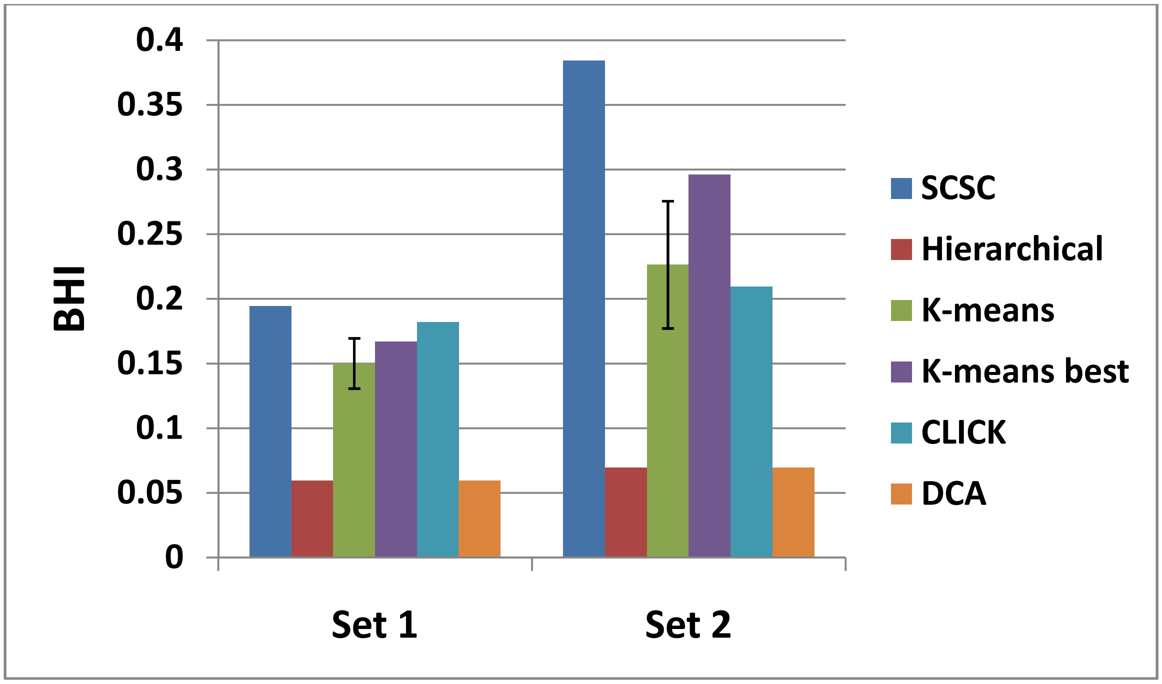

Supplement: Figure S3 — Performance evaluation with independent experimental data. The consistency of a clustering result to a set of co-regulated gene groups is measured by biological homogeneity index (BHI). K-means clustering was run 10 times with different initial values. The red bars and their error bars represent the average BHI and the standard deviation for these 10 runs. The best performance out of the 10 runs is also reported (yellow bar). Two test sets of co-regulated genes groups were defined as follows. Set 1: Co-regulated genes were defined as the genes whose expression levels were affected by all of the seven RNA knockdown (RNAi) experiments of seven regulatory proteins (OCT4, SOX2, NANOG, ESRRB, TBX3, TCL1, DPPA4) which maintain ES cell identity. Each RNAi experiment provided a list of genes whose expression was affected. Taking the intersection of the seven gene lists, a total of 60 genes were identified as regulated by all seven ES cell regulators. These 60 genes were used as the first test set. Set 2: Co-regulated genes were defined as the genes whose expression was affected by RNAi knockdown of all four of the transcription factors NANOG, OCT4, SOX2 and ESRRB, and for which the direction of expression change was the same. These four transcription factors physically interact and synergistically regulate gene expression in ES cells. Two groups of co-regulated genes were identified. Group 1 contained 107 genes that were consistently induced by the RNAi of each of the four factors, whereas group 2 contained 48 genes that were repressed by all four RNAi treatments. These two co-regulated gene groups constitute the second test set. (0.03 MB PDF) [file pcbi.1000707.s007.pdf]
