# Supplementary material for: Modeling Co-Expression across Species for Complex Traits: Insights to the Difference of Human and Mouse Embryonic Stem Cells
Source: PLoS Comput Biol. 2010 Mar 12;6(3):e1000707. doi: 10.1371/journal.pcbi.1000707 (PMC2837392; doi:10.1371/journal.pcbi.1000707)

Figure S4. Scheme of computational implementation of the SCSC method.
The scheme mimics an EM algorithm for clustering one-species data under a Gaussian-mixture model. (Supplementary Document 4)

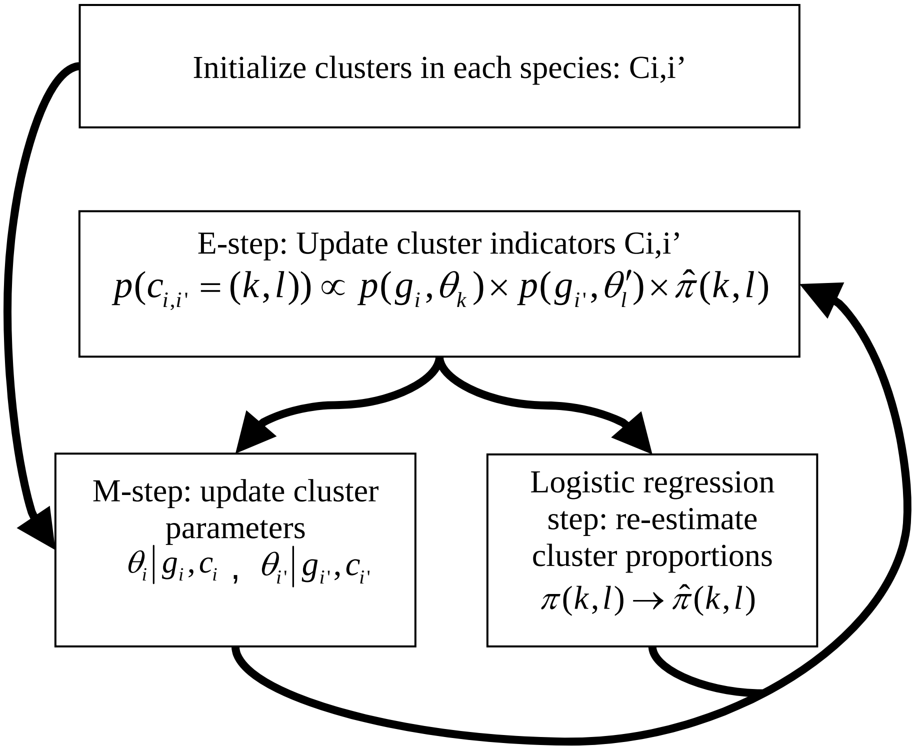

Supplement: Figure S4 — Scheme of computational implementation of the SCSC method. The scheme mimics an EM algorithm for clustering one-species data under a Gaussian-mixture model. (Text S4) (0.02 MB PDF) [file pcbi.1000707.s008.pdf]
