# Supplementary material for: Modeling Co-Expression across Species for Complex Traits: Insights to the Difference of Human and Mouse Embryonic Stem Cells
Source: PLoS Comput Biol. 2010 Mar 12;6(3):e1000707. doi: 10.1371/journal.pcbi.1000707 (PMC2837392; doi:10.1371/journal.pcbi.1000707)
Supplement: Table S1 — Synthetic datasets. Cluster number is the number of clusters in the two species. For example, means 10 clusters in both species. Dimension is the number of samples in each species. “# of data points in each cluster” is the number of orthologous genes in each cluster”. “# of scatter data points” is the number of randomly distributed gene pairs that do not belong to any clusters. They represent intrinsic deviation of the transcriptome from a clustering model. The cluster means of dataset 1–5 are randomly generated between 0 and 10. The cluster mean of dataset 6 are generated between 0 and 13. Cluster variation shows the standard deviation used to generate each cluster, with the two numbers representing two standard deviations for each of the two species. (0.02 MB PDF) [file pcbi.1000707.s009.pdf]

Supplementary Tables

Table S1: Synthetic datasets. Cluster number is the number of clusters in the two species. For example, means 10×10 10 clusters in both species. Dimension is the number of samples in each species. “# of data points in each cluster” is the number of orthologous genes in each cluster”. “# of scatter data points” is the number of randomly distributed gene pairs that do not belong to any clusters. They represent intrinsic deviation of the transcriptome from a clustering model. The cluster means of dataset 1-5 are randomly generated between 0 and 10. The cluster mean of dataset 6 are generated between 0 and 13. Cluster variation shows the standard deviation used to generate each cluster, with the two numbers representing two standard deviations for each of the two species.

Dataset	Cluster number	Dimension	# of data points in each cluster	# of scatter data points	Cluster variation (standard deviation)
1	10×10	10	200	275	[0.8, 0.8]
2	10×10	10	200	275	[0.1, 1.4]
3	10×10	20	200	150	[0.8, 0.8]
4	10×10	20	200	50	[0.8, 0.8]
5	20×20	10	200	150	[0.1, 1.4]
6	4×4	10	320	500	[1.5, 1.5]
