# Supplementary material for: Modeling Co-Expression across Species for Complex Traits: Insights to the Difference of Human and Mouse Embryonic Stem Cells
Source: PLoS Comput Biol. 2010 Mar 12;6(3):e1000707. doi: 10.1371/journal.pcbi.1000707 (PMC2837392; doi:10.1371/journal.pcbi.1000707)
Supplement: Table S2 — Performance evaluation with errors in ortholog map. 10%–30% of the ortholog mapping in synthetic dataset 1 (Table S1) are randomly permutated to represent the scenarios of errors in ortholog map. SCSC, K-means and DCA were executed on these perturbed datasets, and performance metrics were recorded. (0.01 MB PDF) [file pcbi.1000707.s010.pdf]

Table S2. Performance evaluation with errors in ortholog map. 10%-30% of the ortholog mapping in synthetic dataset 1 (Table S1) are randomly permutated to represent the scenarios of errors in ortholog map. SCSC, K-means and DCA were executed on these perturbed datasets, and performance metrics were recorded.

Error proportion in ortholog map	Center scatter			Global scatter			Wrong proportion		
	**SCSC**	**K-means**	**DCA**	**SCSC**	**K-means**	**DCA**	**SCSC**	**K-means**	**DCA**
0%	**1.05**	2.32	1.66	**0.36**	2.07	5.60	**0.07**	0.33	0.80
10%	**1.27**	2.16	4.19	**0.48**	2.50	6.72	**0.09**	0.36	0.90
20%	**1.51**	2.34	4.19	**1.04**	2.84	6.72	**0.16**	0.40	0.90
30%	**1.61**	2.41	4.19	**1.24**	3.08	6.72	**0.19**	0.43	0.90
