# Supplementary material for: Modeling Co-Expression across Species for Complex Traits: Insights to the Difference of Human and Mouse Embryonic Stem Cells
Source: PLoS Comput Biol. 2010 Mar 12;6(3):e1000707. doi: 10.1371/journal.pcbi.1000707 (PMC2837392; doi:10.1371/journal.pcbi.1000707)
Supplement: Table S3 — SCSC clusters. (A) with conserved upregulation in hES and mES cells, (B) specifically upregulated in mES cells, and (C) specifically upregulated in hES cells. (0.15 MB PDF) [file pcbi.1000707.s011.pdf]

Table S3: SCSC clusters (A) with conserved upregulation in hES and mES cells, (B) specifically upregulated in mES cells, and (C) specifically upregulated in hES cells.

A

Cluster	Symbol	Gene description
(2,3)	Whsc2	Wolf-Hirschhorn syndrome candidate 2 (human)
(2,3)	Raf1	v-raf-leukemia viral oncogene 1
(2,3)	Vsnl1	visinin-like 1
(2,3)	Vps72	vacuolar protein sorting 72 (yeast)
(2,3)	Utf1	undifferentiated embryonic cell transcription factor 1
(2,3)	Tyro3	TYRO3 protein tyrosine kinase 3
(2,3)	Trim13	tripartite motif protein 13
(2,3)	Tomm7	translocase of outer mitochondrial membrane 7 homolog (yeast)
(2,3)	Tcea1	transcription elongation factor A (SII) 1
(2,3)	Tgif	TG interacting factor
(2,3)	Tera	teratocarcinoma expressed, serine rich
(2,3)	Smarcd1	SWI/SNF related, matrix associated, actin dependent regulator of chromatin, subfamily d, member 1
(2,3)	Suv39h2	suppressor of variegation 3-9 homolog 2 (Drosophila)
(2,3)	Sub1	SUB1 homolog (S. cerevisia
(2,3)	Spry4	sprouty homolog 4 (Drosophila)
(2,3)	Slc7a8	solute carrier family 7 (cationic amino acid transporter, y+ system), member 8
(2,3)	Sap30	sin3 associated polypeptide
(2,3)	Sh3gl2	SH3-domain GRB2-like 2
(2,3)	Srpk1	serine/arginine-rich protein specific kinase 1
(2,3)	Sema6a	sema domain, transmembrane domain (TM), and cytoplasmic domain, (semaphorin) 6A
(2,3)	Sema3a	sema domain, immunoglobulin domain (Ig), short basic domain, secreted, (semaphorin) 3A
(2,3)	Rwdd1	RWD domain containing 1
(2,3)	Rbpms	RNA binding protein gene with multiple splicing
(2,3)	4931406I20Rik	RIKEN cDNA 4931406I20 gene
(2,3)	Ror1	receptor tyrosine kinase-like orphan receptor 1
(2,3)	Ranbp1	RAN binding protein 1
(2,3)	Rabggtb	RAB geranylgeranyl transferase, b subunit
(2,3)	Pus1	pseudouridine synthase 1
(2,3)	Pim2	proviral integration site 2
(2,3)	Col9a3	procollagen, type IX, alpha 3
(2,3)	Pou2f1	POU domain, class 2, transcription factor 1
(2,3)	Pole3	polymerase (DNA directed), epsilon 3 (p17 subunit)
(2,3)	Pttg1	pituitary tumor-transforming 1
(2,3)	Prps1	phosphoribosyl pyrophosphate synthetase 1
(2,3)	Pltp	phospholipid transfer protein
(2,3)	Pck2	phosphoenolpyruvate carboxykinase 2 (mitochondrial)
(2,3)	Pdlim1	PDZ and LIM domain 1 (elfin)
(2,3)	Nup62	nucleoporin 62

(2,3)	Nfyb	nuclear transcription factor-Y beta
(2,3)	Nanog	Nanog homeobox
(2,3)	Ndufb5	NADH dehydrogenase (ubiquinone) 1 beta subcomplex, 5
(2,3)	Mtmr9	myotubularin related protein 9
(2,3)	Mybl2	myeloblastosis oncogene-like 2
(2,3)	Mrps28	mitochondrial ribosomal protein S28
(2,3)	Mrpl34	mitochondrial ribosomal protein L34
(2,3)	Mcm10	minichromosome maintenance deficient 10 (S. cerevisiae)
(2,3)	Mlx	MAX-like protein X
(2,3)	Mad2l1bp	MAD2L1 binding protein
(2,3)	Lrig1	leucine-rich repeats and immunoglobulin-like domains 1
(2,3)	Lmnb2	lamin B2
(2,3)	Ldhb	lactate dehydrogenase B
(2,3)	Jarid2	jumonji, AT rich interactive domain 2
(2,3)	Ifrd2	interferon-related developmental regulator 2
(2,3)	Imp3	IMP3, U3 small nucleolar ribonucleoprotein, homolog (yeast)
(2,3)	Hesx1	homeo box gene expressed in ES cells
(2,3)	Hmga1	high mobility group AT-hook 1
(2,3)	Hnrpl	heterogeneous nuclear ribonucleoprotein L
(2,3)	Hsp90aa1	heat shock protein 90kDa alpha (cytosolic), class A member 1
(2,3)	Hrk	harakiri, BCL2 interacting protein (contains only BH3 domain)
(2,3)	Grhpr	glyoxylate reductase/hydroxypyruvate reductase
(2,3)	Glo1	glyoxalase 1
(2,3)	Gfpt2	glutamine fructose-6-phosphate transaminase 2
(2,3)	Gnpda1	glucosamine-6-phosphate deaminase 1
(2,3)	Gm288	gene model 288, (NCBI)
(2,3)	Gal	galanin
(2,3)	Fzd7	frizzled homolog 7 (Drosophila)
(2,3)	Fkbp4	FK506 binding protein 4
(2,3)	Fgf2	fibroblast growth factor 2
(2,3)	Daxx	Fas death domain-associated protein
(2,3)	Eif3s7	eukaryotic translation initiation factor 3, subunit 7 (zeta)
(2,3)	Eif2c2	eukaryotic translation initiation factor 2C, 2
(2,3)	Dscr2	Down syndrome critical region homolog 2 (human)
(2,3)	Dnaja3	DnaJ (Hsp40) homolog, subfamily A, member 3
(2,3)	Dnmt1	DNA methyltransferase (cytosine-5) 1
(2,3)	Cox7c	cytochrome c oxidase, subunit VIIc
(2,3)	Ccbl2	cysteine conjugate-beta lyase 2
(2,3)	Cutl2	cut-like 2 (Drosophila)
(2,3)	Crsp2	Cofactor required for Sp1 transcriptional activation, subunit 2
(2,3)	Cstf1	cleavage stimulation factor, 3' pre-RNA, subunit 1
(2,3)	Cldn3	claudin 3
(2,3)	Chga	chromogranin A
(2,3)	Cdk2ap1	CDK2 (cyclin-dependent kinase 2)-associated protein 1
(2,3)	Csnk2b	casein kinase 2, beta polypeptide

(2,3)	Bub1	budding uninhibited by benzimidazoles 1 homolog (S. cerevisiae)
(2,3)	Bckdhb	branched chain ketoacid dehydrogenase E1, beta polypeptide
(2,3)	Aurka	aurora kinase A
(2,3)	Asrgl1	asparaginase like 1
(2,3)	Aass	aminoadipate-semialdehyde synthase
(2,3)	Aldh18a1	aldehyde dehydrogenase 18 family, member A1
(2,3)	Ak2	adenylate kinase 2
(2,3)	Acot8	acyl-CoA thioesterase 8
(2,3)	Acot7	acyl-CoA thioesterase 7
(2,3)	Ascc1	activating signal cointegrator 1 complex subunit 1
(2,3)	Bmpr1b	bone morphogenetic protein receptor, type 1B
(3,3)	Zfp64	zinc finger protein 64
(3,3)	Xrcc5	X-ray repair complementing defective repair in Chinese hamster cells 5
(3,3)	Mycn	v-myc myelocytomatosis viral related oncogene, neuroblastoma derived (avian)
(3,3)	Utp14a	UTP14, U3 small nucleolar ribonucleoprotein, homolog A (yeast)
(3,3)	Uck2	uridine-cytidine kinase 2
(3,3)	Uble1b	ubiquitin-like 1 (sentrin) activating enzyme E1B
(3,3)	Ubap2	ubiquitin-associated protein 2
(3,3)	Usp5	ubiquitin specific peptidase 5 (isopeptidase T)
(3,3)	Tnfsf11	tumor necrosis factor (ligand) superfamily, member 11
(3,3)	Tbca	tubulin cofactor a
(3,3)	Ttk	Ttk protein kinase
(3,3)	Tsr1	TSR1, 20S rRNA accumulation, homolog (yeast)
(3,3)	Tnpo3	transportin 3
(3,3)	Tnpo1	transportin 1
(3,3)	Tomm20	translocase of outer mitochondrial membrane 20 homolog (yeast)
(3,3)	Timm8a1	translocase of inner mitochondrial membrane 8 homolog a1 (yeast)
(3,3)	Tacc3	transforming, acidic coiled-coil containing protein 3
(3,3)	Tars	threonyl-tRNA synthetase
(3,3)	Txnl1	thioredoxin-like 1
(3,3)	Terf1	telomeric repeat binding factor 1
(3,3)	Taf1c	TATA box binding protein (Tbp)-associated factor, RNA polymerase I, C
(3,3)	Supv3l1	suppressor of var1, 3-like 1 (S. cerevisiae)
(3,3)	Supt4h1	suppressor of Ty 4 homolog 1 (S. cerevisiae)
(3,3)	Sox2	SRY-box containing gene 2
(3,3)	Sms	spermine synthase
(3,3)	Slc7a11	solute carrier family 7 (cationic amino acid transporter, y+ system), member 11
(3,3)	Slc29a1	solute carrier family 29 (nucleoside transporters), member 1
(3,3)	Ahcy	S-adenosylhomocysteine hydrolase
(3,3)	Ruvbl1	RuvB-like protein 1
(3,3)	Rrn3	RRN3 RNA polymerase I transcription factor homolog (yeast)
(3,3)	Rbm4	RNA binding motif protein 4
(3,3)	Rbm14	RNA binding motif protein 14
(3,3)	Rnf138	ring finger protein 138
(3,3)	5730410I19Rik	RIKEN cDNA 5730410I19 gene

(3,3)	2810422O20Rik	RIKEN cDNA 2810422O20 gene
(3,3)	2600005C20Rik	RIKEN cDNA 2600005C20 gene
(3,3)	2310057D15Rik	RIKEN cDNA 2310057D15 gene
(3,3)	2010309E21Rik	RIKEN cDNA 2010309E21 gene
(3,3)	Rpe	ribulose-5-phosphate-3-epimerase
(3,3)	Ran	RAN, member RAS oncogene family
(3,3)	Rad51	RAD51 homolog (S. cerevisiae)
(3,3)	Ppa1	pyrophosphatase (inorganic) 1
(3,3)	Prkdc	protein kinase, DNA activated, catalytic polypeptide
(3,3)	Prep	prolyl endopeptidase
(3,3)	Pa2g4	proliferation-associated 2G4
(3,3)	Pou5f1	POU domain, class 5, transcription factor 1
(3,3)	Pola1	polymerase (DNA directed), alpha 1
(3,3)	Phc1	polyhomeotic-like 1 (Drosophila)
(3,3)	Pabpc4	poly A binding protein, cytoplasmic 4
(3,3)	Psat1	phosphoserine aminotransferase 1
(3,3)	Ppat	phosphoribosyl pyrophosphate amidotransferase
(3,3)	Orc5l	origin recognition complex, subunit 5-like (S. cerevisiae)
(3,3)	Nup93	nucleoporin 93
(3,3)	Nup107	nucleoporin 107
(3,3)	Nsd1	nuclear receptor-binding SET-domain protein 1
(3,3)	Myst2	MYST histone acetyltransferase 2
(3,3)	Msh2	mutS homolog 2 (E. coli)
(3,3)	Mtf2	metal response element binding transcription factor 2
(3,3)	Lrpprc	leucine-rich PPR-motif containing
(3,3)	Lmnb1	lamin B1
(3,3)	Kpna1	karyopherin (importin) alpha 1
(3,3)	Itpk1	inositol 1,3,4-triphosphate 5/6 kinase
(3,3)	Hprt1	hypoxanthine guanine phosphoribosyl transferase 1
(3,3)	Hus1	Hus1 homolog (S. pombe)
(3,3)	Hip2	huntingtin interacting protein 2
(3,3)	Hcfc1	host cell factor C1
(3,3)	Hint1	histidine triad nucleotide binding protein 1
(3,3)	Hspa9a	heat shock protein 9A
(3,3)	Gmfb	glia maturation factor, beta
(3,3)	Gspt1	G1 to S phase transition 1
(3,3)	Fkbp3	FK506 binding protein 3
(3,3)	Fem1b	feminization 1 homolog b (C. elegans)
(3,3)	Fastkd5	FAST kinase domains 5
(3,3)	Faf1	Fas-associated factor 1
(3,3)	C80913	expressed sequence C80913
(3,3)	Etv4	ets variant gene 4 (E1A enhancer binding protein, E1AF)
(3,3)	Epb4.1l2	Erythrocyte protein band 4.1-like 2
(3,3)	Eed	embryonic ectoderm development
(3,3)	Ect2	ect2 oncogene

(3,3)	E2f8	E2F transcription factor 8
(3,3)	D19Bwg1357e	DNA segment, Chr 19, Brigham & Women's Genetics 1357 expressed
(3,3)	D15Ertd682e	DNA segment, Chr 15, ERATO Doi 682, expressed
(3,3)	D14Ertd436e	DNA segment, Chr 14, ERATO Doi 436, expressed
(3,3)	Ga17	dendritic cell protein GA17
(3,3)	Ddx54	DEAD (Asp-Glu-Ala-Asp) box polypeptide 54
(3,3)	Ddx27	DEAD (Asp-Glu-Ala-Asp) box polypeptide 27
(3,3)	Ddx18	DEAD (Asp-Glu-Ala-Asp) box polypeptide 18
(3,3)	Ckap2	cytoskeleton associated protein 2
(3,3)	Ctbp2	C-terminal binding protein 2
(3,3)	Cops8	COP9 (constitutive photomorphogenic) homolog, subunit 8 (Arabidopsis thaliana)
(3,3)	Coil	coilin
(3,3)	Chaf1b	chromatin assembly factor 1, subunit B (p60)
(3,3)	Cdc6	cell division cycle 6 homolog (S. cerevisiae)
(3,3)	Cnot7	CCR4-NOT transcription complex, subunit 7
(3,3)	Cacybp	calcyclin binding protein
(3,3)	Bard1	BRCA1 associated RING domain 1
(3,3)	Bet1	blocked early in transport 1 homolog (S. cerevisiae)
(3,3)	Bclaf1	BCL2-associated transcription factor 1
(3,3)	Btf3	basic transcription factor 3
(3,3)	Dars	aspartyl-tRNA synthetase
(3,3)	Asns	asparagine synthetase
(3,3)	Asf1a	ASF1 anti-silencing function 1 homolog A (S. cerevisiae)
(3,3)	Ars2	arsenate resistance protein 2
(3,3)	Actr6	ARP6 actin-related protein 6 homolog (yeast)
(3,3)	Aqr	aquarius
(3,3)	Apex1	apurinic/apyrimidinic endonuclease 1
(3,3)	Akp2	alkaline phosphatase 2, liver
(3,3)	Akap12	A kinase (PRKA) anchor protein (gravin) 12
(3,3)	Dppa4	developmental pluripotency associated 4
(3,3)	Tdgf1	teratocarcinoma-derived growth factor

C

Cluster	Symbol	Gene description
(1,3)	Twsg1	twisted gastrulation homolog 1 (Drosophila)
(1,3)	Trove2	TROVE domain family, member 2
(1,3)	Tmem70	transmembrane protein 70
(1,3)	Tle1	transducin-like enhancer of split 1, homolog of Drosophila E(spl)
(1,3)	Tcfcp2	transcription factor CP2
(1,3)	Tcf4	transcription factor 4
(1,3)	Top2b	topoisomerase (DNA) II beta
(1,3)	Tmpo	thymopoietin
(1,3)	Spast	spastin
(1,3)	Slc30a5	solute carrier family 30 (zinc transporter), member 5
(1,3)	Stk39	serine/threonine kinase 39, STE20/SPS1 homolog (yeast)
(1,3)	Sec63	SEC63-like (S. cerevisiae)
(1,3)	Sar1a	SAR1 gene homolog A (S. cerevisiae)
(1,3)	Robo1	roundabout homolog 1 (Drosophila)
(1,3)	Rprm	reprimo, TP53 dependent G2 arrest mediator candidate
(1,3)	Rit2	Ras-like without CAAX 2
(1,3)	Rab28	RAB28, member RAS oncogene family
(1,3)	Rabgap1l	RAB GTPase activating protein 1-like
(1,3)	Prkcbp1	protein kinase C binding protein 1
(1,3)	Prepl	prolyl endopeptidase-like
(1,3)	Pbx3	pre B-cell leukemia transcription factor 3
(1,3)	Kcnd2	potassium voltage-gated channel, Shal-related family, member 2
(1,3)	Plekhc1	pleckstrin homology domain containing, family C (with FERM domain) member 1
(1,3)	Pafah1b1	platelet-activating factor acetylhydrolase, isoform 1b, beta1 subunit
(1,3)	Peg3	paternally expressed 3
(1,3)	Nfe2l3	nuclear factor, erythroid derived 2, like 3
(1,3)	Npy1r	neuropeptide Y receptor Y1
(1,3)	Mpdz	multiple PDZ domain protein
(1,3)	Mapre1	microtubule-associated protein, RP/EB family, member 1
(1,3)	Mest	mesoderm specific transcript
(1,3)	Magel2	melanoma antigen, family L, 2
(1,3)	Man2a1	mannosidase 2, alpha 1
(1,3)	Large	like-glycosyltransferase
(1,3)	Kif1a	kinesin family member 1A
(1,3)	Klhl7	kelch-like 7 (Drosophila)
(1,3)	Itsn1	intersectin 1 (SH3 domain protein 1A)
(1,3)	Gap43	growth associated protein 43
(1,3)	Gpm6b	glycoprotein m6b
(1,3)	Flt1	FMS-like tyrosine kinase 1
(1,3)	AW146242	expressed sequence AW146242
(1,3)	Enpp1	ectonucleotide pyrophosphatase/phosphodiesterase 1
(1,3)	Dnm1	dynamin 1

(1,3)	Dpm1	dolichol-phosphate (beta-D) mannosyltransferase 1
(1,3)	D0H4S114	DNA segment, human D4S114
(1,3)	Dpysl3	dihydropyrimidinase-like 3
(1,3)	Cri1	CREBBP/EP300 inhibitory protein 1
(1,3)	Clu	clusterin
(1,3)	Cstf2t	cleavage stimulation factor, 3' pre-RNA subunit 2, tau
(1,3)	Clic4	chloride intracellular channel 4 (mitochondrial)
(1,3)	Cenpe	centromere protein E
(1,3)	Centg2	centaurin, gamma 2
(1,3)	Cdc16	CDC16 cell division cycle 16 homolog (S. cerevisiae)
(1,3)	Csnk1d	casein kinase 1, delta
(1,3)	Alg5	asparagine-linked glycosylation 5 homolog (yeast, dolichyl-phosphate beta-glucosyltransferase)
(1,3)	Armcx2	armadillo repeat containing, X-linked 2
(1,3)	Rere	arginine glutamic acid dipeptide (RE) repeats
(1,3)	Ank3	ankyrin 3, epithelial
(1,3)	Appbp1	amyloid beta precursor protein binding protein 1
(1,3)	Pdpk1	3-phosphoinositide dependent protein kinase-1
(4,3)	Wrn	Werner syndrome homolog (human)
(4,3)	Umps	uridine monophosphate synthetase
(4,3)	Unc13b	unc-13 homolog B (C. elegans)
(4,3)	Tbl1x	Transducin (beta)-like 1 X-linked
(4,3)	Tfam	transcription factor A, mitochondrial
(4,3)	Tcf7l2	transcription factor 7-like 2, T-cell specific, HMG-box
(4,3)	Taf1b	TATA box binding protein (Tbp)-associated factor, RNA polymerase I, B
(4,3)	Taf6	TAF6 RNA polymerase II, TATA box binding protein (TBP)-associated factor
(4,3)	Syt1	synaptotagmin I
(4,3)	Stc1	stanniocalcin 1
(4,3)	Sart3	squamous cell carcinoma antigen recognized by T-cells 3
(4,3)	Srp54	signal recognition particle 54
(4,3)	Rbm9	RNA binding motif protein 9
(4,3)	Rbm15	RNA binding motif protein 15
(4,3)	Ptpn11	protein tyrosine phosphatase, non-receptor type 11
(4,3)	Pbx1	pre B-cell leukemia transcription factor 1
(4,3)	Kcnq2	potassium voltage-gated channel, subfamily Q, member 2
(4,3)	Pctk3	PCTAIRE-motif protein kinase 3
(4,3)	Nell2	NEL-like 2 (chicken)
(4,3)	Nrd1	nardilysin, N-arginine dibasic convertase, NRD convertase 1
(4,3)	Gnptab	N-acetylglucosamine-1-phosphate transferase, alpha and beta subunits
(4,3)	Ms4a2	membrane-spanning 4-domains, subfamily A, member 2
(4,3)	Lrp6	low density lipoprotein receptor-related protein 6
(4,3)	Ldb2	LIM domain binding 2
(4,3)	Larp7	La ribonucleoprotein domain family, member 7
(4,3)	Indo	indoleamine-pyrrole 2,3 dioxygenase
(4,3)	Fgfr2	fibroblast growth factor receptor 2

(4,3)	Epb4.1l1	erythrocyte protein band 4.1-like 1
(4,3)	D2Wsu81e	DNA segment, Chr 2, Wayne State University 81, expressed
(4,3)	Cxadr	coxsackievirus and adenovirus receptor
(4,3)	Bmp4	bone morphogenetic protein 4
(4,3)	Auh	AU RNA binding protein/enoyl-coenzyme A hydratase
(4,3)	Aldh1a2	aldehyde dehydrogenase family 1, subfamily A2
(4,3)	Akap1	A kinase (PRKA) anchor protein 1
(5,3)	Zzz3	zinc finger, ZZ domain containing 3
(5,3)	Zfml	zinc finger, matrin-like
(5,3)	Zfp281	zinc finger protein 281
(5,3)	Kras	v-Ki-ras2 Kirsten rat sarcoma viral oncogene homolog
(5,3)	Vcl	vinculin
(5,3)	Vdp	vesicle docking protein
(5,3)	Vrk1	vaccinia related kinase 1
(5,3)	Ugdh	UDP-glucose dehydrogenase
(5,3)	Ube2g1	ubiquitin-conjugating enzyme E2G 1 (UBC7 homolog, C. elegans)
(5,3)	Usp1	ubiquitin specific peptdiase 1
(5,3)	---	U63720:Mus musculus CPP32 apoptotic protease mRNA
(5,3)	Tnfrsf11a	tumor necrosis factor receptor superfamily, member 11a
(5,3)	Tmeff1	transmembrane protein with EGF-like and two follistatin-like domains 1
(5,3)	Trpc1	transient receptor potential cation channel, subfamily C, member 1
(5,3)	Tfrc	transferrin receptor
(5,3)	Tceb1	transcription elongation factor B (SIII), polypeptide 1
(5,3)	Topbp1	topoisomerase (DNA) II beta binding protein
(5,3)	Tbpl1	TATA box binding protein-like 1
(5,3)	Tardbp	TAR DNA binding protein
(5,3)	Syncrip	synaptotagmin binding, cytoplasmic RNA interacting protein
(5,3)	Smarca5	SWI/SNF related, matrix associated, actin dependent regulator of chromatin, subfamily a, member 5
(5,3)	Smc4	structural maintenance of chromosomes 4
(5,3)	Smc2	structural maintenance of chromosomes 2
(5,3)	Sfrs3	splicing factor, arginine/serine-rich 3 (SRp20)
(5,3)	Sfrs2	splicing factor, arginine/serine-rich 2 (SC-35)
(5,3)	Slc33a1	solute carrier family 33 (acetyl-CoA transporter), member 1
(5,3)	Sumo1	SMT3 suppressor of mif two 3 homolog 1 (yeast)
(5,3)	Ssb	Sjogren syndrome antigen B
(5,3)	Sap18	Sin3-associated polypeptide 18
(5,3)	Serbp1	Serpine1 mRNA binding protein 1
(5,3)	Rrs1	RRS1 ribosome biogenesis regulator homolog (S. cerevisiae)
(5,3)	4921531G14Rik	RIKEN cDNA 4921531G14 gene
(5,3)	3300001P08Rik	RIKEN cDNA 3300001P08 gene
(5,3)	2810405J04Rik	RIKEN cDNA 2810405J04 gene
(5,3)	Rpn2	ribophorin II
(5,3)	Rcbtb1	regulator of chromosome condensation (RCC1) and BTB (POZ) domain containing protein 1
(5,3)	R3hdm1	R3H domain 1 (binds single-stranded nucleic acids)

(5,3)	Qk	quaking
(5,3)	Ptk2	PTK2 protein tyrosine kinase 2
(5,3)	Prpf4b	PRP4 pre-mRNA processing factor 4 homolog B (yeast)
(5,3)	Ptprk	protein tyrosine phosphatase, receptor type, K
(5,3)	Ptprg	protein tyrosine phosphatase, receptor type, G
(5,3)	Ppm1b	protein phosphatase 1B, magnesium dependent, beta isoform
(5,3)	Ppp1cc	protein phosphatase 1, catalytic subunit, gamma isoform
(5,3)	Psme4	proteasome (prosome, macropain) activator subunit 4
(5,3)	Pgrmc1	progesterone receptor membrane component 1
(5,3)	Parg	poly (ADP-ribose) glycohydrolase
(5,3)	Pkp4	plakophilin 4
(5,3)	Prdx3	peroxiredoxin 3
(5,3)	Ppid	peptidylprolyl isomerase D (cyclophilin D)
(5,3)	Ptch1	patched homolog 1
(5,3)	Otud4	OTU domain containing 4
(5,3)	Nsbp1	nucleosome binding protein 1
(5,3)	Nup50	nucleoporin 50
(5,3)	Nucks1	nuclear casein kinase and cyclin-dependent kinase substrate 1
(5,3)	Ncbp2	nuclear cap binding protein subunit 2
(5,3)	Narg1	NMDA receptor-regulated gene 1
(5,3)	Nlk	nemo like kinase
(5,3)	Ndufs1	NADH dehydrogenase (ubiquinone) Fe-S protein 1
(5,3)	Map3k7	mitogen activated protein kinase kinase kinase 7
(5,3)	Mtif2	mitochondrial translational initiation factor 2
(5,3)	Metap1	methionyl aminopeptidase 1
(5,3)	Matr3	matrin 3
(5,3)	Smad2	MAD homolog 2 (Drosophila)
(5,3)	Kif2a	Kinesin family member 2A
(5,3)	Khdrbs1	KH domain containing, RNA binding, signal transduction associated 1
(5,3)	Kpnb1	karyopherin (importin) beta 1
(5,3)	Jarid1b	jumonji, AT rich interactive domain 1B (Rbp2 like)
(5,3)	Iars2	isoleucine-tRNA synthetase 2, mitochondrial
(5,3)	Impact	imprinted and ancient
(5,3)	Htatsf1	HIV TAT specific factor 1
(5,3)	Hmgb1	high mobility group box 1
(5,3)	Hnrpr	heterogeneous nuclear ribonucleoprotein R
(5,3)	Hnrpa2b1	heterogeneous nuclear ribonucleoprotein A2/B1
(5,3)	Hs2st1	heparan sulfate 2-O-sulfotransferase 1
(5,3)	Hspa4	heat shock protein 4
(5,3)	Gatad2a	GATA zinc finger domain containing 2A
(5,3)	Gja1	gap junction membrane channel protein alpha 1
(5,3)	Fzd3	frizzled homolog 3 (Drosophila)
(5,3)	Etv1	ets variant gene 1
(5,3)	Ei24	etoposide induced 2.4 mRNA
(5,3)	Ergic2	ERGIC and golgi 2

(5,3)	Eny2	enhancer of yellow 2 homolog (Drosophila)
(5,3)	E2f1	E2F transcription factor 1
(5,3)	Dtl	denticleless homolog (Drosophila)
(5,3)	Dek	DEK oncogene (DNA binding)
(5,3)	Dhx9	DEAH (Asp-Glu-Ala-His) box polypeptide 9
(5,3)	Cul3	cullin 3
(5,3)	Cul1	cullin 1
(5,3)	Cltc	clathrin, heavy polypeptide (Hc)
(5,3)	Chd4	chromodomain helicase DNA binding protein 4
(5,3)	Cct8	chaperonin subunit 8 (theta)
(5,3)	Cetn3	centrin 3
(5,3)	Cnbp	cellular nucleic acid binding protein
(5,3)	Cdc7	cell division cycle 7 (S. cerevisiae)
(5,3)	Casp8ap2	caspase 8 associated protein 2
(5,3)	Csnk2a1	casein kinase 2, alpha 1 polypeptide
(5,3)	Atg12	autophagy-related 12 (yeast)
(5,3)	Angel2	angel homolog 2 (Drosophila)
(5,3)	Adss	adenylosuccinate synthetase, non muscle
(5,3)	Adcy2	adenylate cyclase 2
(6,3)	Zwint	ZW10 interactor
(6,3)	Zdhhc6	zinc finger, DHHC domain containing 6
(6,3)	Zic3	zinc finger protein of the cerebellum 3
(6,3)	Zfp143	zinc finger protein 143
(6,3)	Yy1	YY1 transcription factor
(6,3)	Galnt11	UDP-N-acetyl-alpha-D-galactosamine:polypeptide N-acetylgalactosaminyltransferase 11
(6,3)	Trp53bp1	transformation related protein 53 binding protein 1
(6,3)	Tgif2	TGFB-induced factor 2
(6,3)	Tgds	TDP-glucose 4,6-dehydratase
(6,3)	Tbp	TATA box binding protein
(6,3)	Sdf2	stromal cell derived factor 2
(6,3)	Stc2	stanniocalcin 2
(6,3)	Sfrs9	splicing factor, arginine/serine rich 9
(6,3)	Sri	sorcin
(6,3)	Son	Son cell proliferation protein
(6,3)	Slc30a9	solute carrier family 30 (zinc transporter), member 9
(6,3)	Slc16a1	solute carrier family 16 (monocarboxylic acid transporters), member 1
(6,3)	Rnf5	ring finger protein 5
(6,3)	9130211I03Rik	RIKEN cDNA 9130211I03 gene
(6,3)	1600012H06Rik	RIKEN cDNA 1600012H06 gene
(6,3)	Ppp2r5e	Protein phosphatase 2, regulatory subunit B (B56), epsilon isoform
(6,3)	Podxl	podocalyxin-like
(6,3)	Plekha5	pleckstrin homology domain containing, family A member 5
(6,3)	Nr2c1	nuclear receptor subfamily 2, group C, member 1
(6,3)	Minpp1	multiple inositol polyphosphate histidine phosphatase 1

(6,3)	Mcam	melanoma cell adhesion molecule
(6,3)	Laptm4b	lysosomal-associated protein transmembrane 4B
(6,3)	Lass2	longevity assurance homolog 2 (S. cerevisiae)
(6,3)	Hdac2	histone deacetylase 2
(6,3)	Grsf1	G-rich RNA sequence binding factor 1
(6,3)	Gpc4	glypican 4
(6,3)	Gtf2e2	general transcription factor II E, polypeptide 2 (beta subunit)
(6,3)	Frat2	frequently rearranged in advanced T-cell lymphomas 2
(6,3)	Fbxo21	F-box only protein 21
(6,3)	Erp29	endoplasmic reticulum protein 29
(6,3)	E2f5	E2F transcription factor 5
(6,3)	Ddx6	DEAD (Asp-Glu-Ala-Asp) box polypeptide 6
(6,3)	Ccnc	cyclin C
(6,3)	Ccdc99	coiled-coil domain containing 99
(6,3)	Coch	coagulation factor C homolog (Limulus polyphemus)
(6,3)	Cbx5	chromobox homolog 5 (Drosophila HP1a)
(6,3)	Cbx3	chromobox homolog 3 (Drosophila HP1 gamma)
(6,3)	Clk2	CDC-like kinase 2
(6,3)	Ctsc	cathepsin C
(6,3)	Ctnna1	catenin (cadherin associated protein), alpha 1
(6,3)	Calca	calcitonin/calcitonin-related polypeptide, alpha
(6,3)	Bub3	budding uninhibited by benzimidazoles 3 homolog (S. cerevisiae)
(6,3)	St6gal1	beta galactoside alpha 2,6 sialyltransferase 1
(6,3)	Ankrd46	ankyrin repeat domain 46
(6,3)	Aldh7a1	aldehyde dehydrogenase family 7, member A1
(6,3)	Acvr2b	activin receptor IIB
(6,3)	Anp32a	acidic (leucine-rich) nuclear phosphoprotein 32 family, member A

B

Cluster	Symbol	Gene description
(2,1)	Zfpn1a1	zinc finger protein, subfamily 1A, 1 (Ikaros)
(2,1)	Zfp36l2	zinc finger protein 36, C3H type-like 2
(2,1)	Zap70	zeta-chain (TCR) associated protein kinase
(2,1)	Maff	v-maf musculoaponeurotic fibrosarcoma oncogene family, protein F (avian)
(2,1)	Vdr	vitamin D receptor
(2,1)	Vax2	ventral anterior homeobox containing gene 2
(2,1)	Upp1	uridine phosphorylase 1
(2,1)	Ulk1	Unc-51 like kinase 1 (C. elegans)
(2,1)	Tnk2	tyrosine kinase, non-receptor, 2
(2,1)	Txk	TXK tyrosine kinase
(2,1)	Tnfsf8	tumor necrosis factor (ligand) superfamily, member 8
(2,1)	Tnfsf4	tumor necrosis factor (ligand) superfamily, member 4
(2,1)	Tmc6	transmembrane channel-like gene family 6
(2,1)	Tspo	translocator protein
(2,1)	Tgfbr2	transforming growth factor, beta receptor II
(2,1)	Tgfb1	transforming growth factor, beta 1
(2,1)	Mdm4	transformed mouse 3T3 cell double minute 4
(2,1)	Thrb	thyroid hormone receptor beta
(2,1)	Txnip	thioredoxin interacting protein
(2,1)	Tcl1	T-cell lymphoma breakpoint 1
(2,1)	Tbx2	T-box 2
(2,1)	Tapbp	TAP binding protein
(2,1)	Sfn	stratifin
(2,1)	Sox15	SRY-box containing gene 15
(2,1)	Syk	spleen tyrosine kinase
(2,1)	Slc4a1	solute carrier family 4 (anion exchanger), member 1
(2,1)	Slc25a28	solute carrier family 25, member 28
(2,1)	Slc1a6	solute carrier family 1 (high affinity aspartate/glutamate transporter), member 6
(2,1)	Stat6	signal transducer and activator of transcription 6
(2,1)	Stat5b	signal transducer and activator of transcription 5B
(2,1)	Stat5a	signal transducer and activator of transcription 5A
(2,1)	Stat4	signal transducer and activator of transcription 4
(2,1)	Sfpi1	SFFV proviral integration 1
(2,1)	Rbm38	RNA binding motif protein 38
(2,1)	4632413K17Rik	RIKEN cDNA 4632413K17 gene
(2,1)	2310004I24Rik	RIKEN cDNA 2310004I24 gene
(2,1)	2300002G24Rik	RIKEN cDNA 2300002G24 gene
(2,1)	1190002H23Rik	RIKEN cDNA 1190002H23 gene
(2,1)	1110008P14Rik	RIKEN cDNA 1110008P14 gene
(2,1)	1100001H23Rik	RIKEN cDNA 1100001H23 gene
(2,1)	Rps6ka1	ribosomal protein S6 kinase polypeptide 1

(2,1)	Rarg	retinoic acid receptor, gamma
(2,1)	Rfx2	regulatory factor X, 2 (influences HLA class II expression)
(2,1)	Rasa4	RAS p21 protein activator 4
(2,1)	Rhod	ras homolog gene family, member D
(2,1)	Rap2b	RAP2B, member of RAS oncogene family
(2,1)	Pim1	proviral integration site 1
(2,1)	Psmb9	proteosome (prosome, macropain) subunit, beta type 9 (large multifunctional peptidase 2)
(2,1)	Psmb8	proteosome (prosome, macropain) subunit, beta type 8 (large multifunctional peptidase 7)
(2,1)	Psmb10	proteasome (prosome, macropain) subunit, beta type 10
(2,1)	Ptgdr	prostaglandin D receptor
(2,1)	Pml	promyelocytic leukemia
(2,1)	Pstpip2	proline-serine-threonine phosphatase-interacting protein 2
(2,1)	Pstpip1	proline-serine-threonine phosphatase-interacting protein 1
(2,1)	Kcna3	potassium voltage-gated channel, shaker-related subfamily, member 3
(2,1)	Pscd1	pleckstrin homology, Sec7 and coiled-coil domains 1
(2,1)	Plaur	plasminogen activator, urokinase receptor
(2,1)	Plcg2	phospholipase C, gamma 2
(2,1)	Olfm1	olfactomedin 1
(2,1)	Nr1d1	nuclear receptor subfamily 1, group D, member 1
(2,1)	Ncoa1	nuclear receptor coactivator 1
(2,1)	Nfil3	nuclear factor, interleukin 3, regulated
(2,1)	Nfkb2	nuclear factor of kappa light polypeptide gene enhancer in B-cells 2, p49/p100
(2,1)	Nfkbib	nuclear factor of kappa light chain gene enhancer in B-cells inhibitor, beta
(2,1)	Neurod6	neurogenic differentiation 6
(2,1)	Nedd4l	neural precursor cell expressed, developmentally down-regulated gene 4-like
(2,1)	Mef2b	myocyte enhancer factor 2B
(2,1)	Myb	myeloblastosis oncogene
(2,1)	Muc1	mucin 1, transmembrane
(2,1)	Mgll	monoglyceride lipase
(2,1)	Mapk8ip3	mitogen-activated protein kinase 8 interacting protein 3
(2,1)	Mapk3	mitogen activated protein kinase 3
(2,1)	Mapk13	mitogen activated protein kinase 13
(2,1)	Matk	megakaryocyte-associated tyrosine kinase
(2,1)	Mxd3	Max dimerization protein 3
(2,1)	Mfng	manic fringe homolog (Drosophila)
(2,1)	Mvp	major vault protein
(2,1)	Lyst	lysosomal trafficking regulator
(2,1)	Ltbr	lymphotoxin B receptor
(2,1)	Ltb	lymphotoxin B
(2,1)	Lef1	lymphoid enhancer binding factor 1
(2,1)	Lag3	lymphocyte-activation gene 3
(2,1)	Lck	lymphocyte protein tyrosine kinase
(2,1)	Limd2	LIM domain containing 2
(2,1)	Lgals9	lectin, galactose binding, soluble 9
(2,1)	Lgals3	lectin, galactose binding, soluble 3

(2,1)	Klf5	Kruppel-like factor 5
(2,1)	Klf13	Kruppel-like factor 13
(2,1)	Klk8	kallikrein 8
(2,1)	Junb	Jun-B oncogene
(2,1)	Jund1	Jun proto-oncogene related gene d1
(2,1)	Iqgap1	IQ motif containing GTPase activating protein 1
(2,1)	Il6ra	interleukin 6 receptor, alpha
(2,1)	Il4ra	interleukin 4 receptor, alpha
(2,1)	Il2rg	interleukin 2 receptor, gamma chain
(2,1)	Irf1	interferon regulatory factor 1
(2,1)	Itgb7	integrin beta 7
(2,1)	Itgam	integrin alpha M
(2,1)	Itga4	integrin alpha 4
(2,1)	Hcn2	hyperpolarization-activated, cyclic nucleotide-gated K+ 2
(2,1)	Hic1	hypermethylated in cancer 1
(2,1)	Hoxa4	homeo box A4
(2,1)	Hist2h3c1	histone 2, H3c1
(2,1)	Hist1h2bp	Histone 1, H2bp
(2,1)	Hist1h1e	histone 1, H1e
(2,1)	Hmha1	histocompatibility (minor) HA-1
(2,1)	Hes2	hairy and enhancer of split 2 (Drosophila)
(2,1)	Hr	hairless
(2,1)	H2afj	H2A histone family, member J
(2,1)	Hlx1	H2.0-like homeo box 1 (Drosophila)
(2,1)	Gch1	GTP cyclohydrolase 1
(2,1)	Gab2	growth factor receptor bound protein 2-associated protein 2
(2,1)	Gadd45b	growth arrest and DNA-damage-inducible 45 beta
(2,1)	Grap2	GRB2-related adaptor protein 2
(2,1)	Gstk1	glutathione S-transferase kappa 1
(2,1)	Gjb5	gap junction membrane channel protein beta 5
(2,1)	Gjb3	gap junction membrane channel protein beta 3
(2,1)	Gpr137b	G protein-coupled receptor 137B
(2,1)	Folr1	folate receptor 1 (adult)
(2,1)	Flt3	FMS-like tyrosine kinase 3
(2,1)	Fgfbp1	fibroblast growth factor binding protein 1
(2,1)	Ehf	ets homologous factor
(2,1)	Emcn	endomucin
(2,1)	Elf3	E74-like factor 3
(2,1)	Dync1i1	dynein cytoplasmic 1 intermediate chain 1
(2,1)	Drap1	Dr1 associated protein 1 (negative cofactor 2 alpha)
(2,1)	Dmc1h	disrupted meiotic cDNA 1 homolog
(2,1)	Crlf3	cytokine receptor-like factor 3
(2,1)	Cyba	cytochrome b-245, alpha polypeptide
(2,1)	Crip1	cysteine-rich protein 1 (intestinal)
(2,1)	Csf2ra	colony stimulating factor 2 receptor, alpha, low-affinity (granulocyte-macrophage)

(2,1)	Chchd2	coiled-coil-helix-coiled-coil-helix domain containing 2
(2,1)	Cldn4	claudin 4
(2,1)	Centb1	centaurin, beta 1
(2,1)	Clk3	CDC-like kinase 3
(2,1)	Cdc42se1	CDC42 small effector 1
(2,1)	Cd83	CD83 antigen
(2,1)	Cd8b1	CD8 antigen, beta chain 1
(2,1)	Cd79b	CD79B antigen
(2,1)	Cd40	CD40 antigen
(2,1)	Cd28	CD28 antigen
(2,1)	Comt	catechol-O-methyltransferase
(2,1)	Car12	carbonic anyhydrase 12
(2,1)	Car2	carbonic anhydrase 2
(2,1)	Capg	capping protein (actin filament), gelsolin-like
(2,1)	Blnk	B-cell linker
(2,1)	Aire	autoimmune regulator (autoimmune polyendocrinopathy candidiasis ectodermal dystrophy)
(2,1)	Atn1	atrophin 1
(2,1)	Abcb4	ATP-binding cassette, sub-family B (MDR/TAP), member 4
(2,1)	Abca2	ATP-binding cassette, sub-family A (ABC1), member 2
(2,1)	Atp2a3	ATPase, Ca++ transporting, ubiquitous
(2,1)	Avpi1	arginine vasopressin-induced 1
(2,1)	Avp	arginine vasopressin
(2,1)	Aes	amino-terminal enhancer of split
(2,1)	Alad	aminolevulinate, delta-, dehydratase
(2,1)	Ap1b1	adaptor protein complex AP-1, beta 1 subunit
(2,1)	Ache	acetylcholinesterase
(2,1)	Abhd8	abhydrolase domain containing 8
(2,1)	Agpat3	1-acylglycerol-3-phosphate O-acyltransferase 3
(2,4)	Zyx	zyxin
(2,4)	Zbtb7b	Vzinc finger and BTB domain containing 7B
(2,4)	Vipr2	vasoactive intestinal peptide receptor 2
(2,4)	Tead3	TEA domain family member 3
(2,4)	Taf10	TAF10 RNA polymerase II, TATA box binding protein (TBP)-associated factor
(2,4)	Synj2	synaptojanin 2
(2,4)	Supt6h	suppressor of Ty 6 homolog (S. cerevisiae)
(2,4)	Sdf4	stromal cell derived factor 4
(2,4)	Slc22a5	solute carrier family 22 (organic cation transporter), member 5
(2,4)	Serpine1	serine (or cysteine) peptidase inhibitor, clade E, member 1
(2,4)	Ryr1	ryanodine receptor 1, skeletal muscle
(2,4)	Src	Rous sarcoma oncogene
(2,4)	1500034J01Rik	RIKEN cDNA 1500034J01 gene
(2,4)	1110033J19Rik	RIKEN cDNA 1110033J19 gene
(2,4)	Rbbp4	retinoblastoma binding protein 4
(2,4)	Rac3	RAS-related C3 botulinum substrate 3
(2,4)	Rad51l1	RAD51-like 1 (S. cerevisiae)

(2,4)	Ptprn	protein tyrosine phosphatase, receptor type, N
(2,4)	Ppp1r3c	protein phosphatase 1, regulatory (inhibitor) subunit 3C
(2,4)	Prkar2a	protein kinase, cAMP dependent regulatory, type II alpha
(2,4)	Col8a1	procollagen, type VIII, alpha 1
(2,4)	Kcnma1	potassium large conductance calcium-activated channel, subfamily M, alpha member 1
(2,4)	Pld3	phospholipase D family, member 3
(2,4)	Pip5k1c	phosphatidylinositol-4-phosphate 5-kinase, type 1 gamma
(2,4)	Pxn	paxillin
(2,4)	Prcc	papillary renal cell carcinoma (translocation-associated)
(2,4)	Npdc1	neural proliferation, differentiation and control gene 1
(2,4)	Ndst1	N-deacetylase/N-sulfotransferase (heparan glucosaminyl) 1
(2,4)	Map3k6	mitogen-activated protein kinase kinase kinase 6
(2,4)	Mt2	metallothionein 2
(2,4)	Mmp9	matrix metallopeptidase 9
(2,4)	Matn3	matrilin 3
(2,4)	Mrc2	mannose receptor, C type 2
(2,4)	Lrp5	low density lipoprotein receptor-related protein 5
(2,4)	Lima1	LIM domain and actin binding 1
(2,4)	Lmna	lamin A
(2,4)	Itgb4	integrin beta 4
(2,4)	Inpp1	inositol polyphosphate-1-phosphatase
(2,4)	Hcfc1r1	host cell factor C1 regulator 1 (XPO1-dependent)
(2,4)	Bat2	HLA-B associated transcript 2
(2,4)	Hist1h2bb	histone 1, H2bb
(2,4)	Rras	Harvey rat sarcoma oncogene, subgroup R
(2,4)	Gramd3	GRAM domain containing 3
(2,4)	Cga	glycoprotein hormones, alpha subunit
(2,4)	Glrx	glutaredoxin
(2,4)	G6pd2	glucose-6-phosphate dehydrogenase 2
(2,4)	Fzd9	frizzled homolog 9 (Drosophila)
(2,4)	Freq	frequenin homolog (Drosophila)
(2,4)	Fgf9	fibroblast growth factor 9
(2,4)	Fgf1	fibroblast growth factor 1
(2,4)	Epor	erythropoietin receptor
(2,4)	Egfr	epidermal growth factor receptor
(2,4)	Efna5	ephrin A5
(2,4)	Eng	endoglin
(2,4)	D4Wsu114e	DNA segment, Chr 4, Wayne State University 114, expressed
(2,4)	Dlx4	distal-less homeobox 4
(2,4)	Dtx2	deltex 2 homolog (Drosophila)
(2,4)	Dcc	deleted in colorectal carcinoma
(2,4)	Ddhd2	DDHD domain containing 2
(2,4)	Carm1	coactivator-associated arginine methyltransferase 1
(2,4)	Cnn1	calponin 1
(2,4)	Capns1	calpain, small subunit 1

(2,4)	Celsr1	cadherin EGF LAG seven-pass G-type receptor 1
(2,4)	Bdkrb2	bradykinin receptor, beta 2
(2,4)	Bhmt2	betaine-homocysteine methyltransferase 2
(2,4)	Bik	Bcl2-interacting killer
(2,4)	Atg7	autophagy-related 7 (yeast)
(2,4)	Alox15	arachidonate 15-lipoxygenase
(2,4)	Htr1f	5-hydroxytryptamine (serotonin) receptor 1F
(2,5)	Araf	v-raf murine sarcoma 3611 viral oncogene homolog
(2,5)	Erbb3	v-erb-b2 erythroblastic leukemia viral oncogene homolog 3 (avian)
(2,5)	Unc84a	unc-84 homolog A (C. elegans)
(2,5)	Tnfrsf21	tumor necrosis factor receptor superfamily, member 21
(2,5)	Tulp3	tubby-like protein 3
(2,5)	Trim28	tripartite motif protein 28
(2,5)	Trp53	transformation related protein 53
(2,5)	Sin3b	transcriptional regulator, SIN3B (yeast)
(2,5)	Tcf2	transcription factor 2
(2,5)	Sox13	SRY-box containing gene 13
(2,5)	Spnb3	spectrin beta 3
(2,5)	Sec22b	SEC22 vesicle trafficking protein homolog B (S. cerevisiae)
(2,5)	Sec14l1	SEC14-like 1 (S. cerevisiae)
(2,5)	A430005L14Rik	RIKEN cDNA A430005L14 gene
(2,5)	4932441K18Rik	RIKEN cDNA 4932441K18 gene
(2,5)	2810002I04Rik	RIKEN cDNA 2810002I04 gene
(2,5)	Pink1	PTEN induced putative kinase 1
(2,5)	Ptpn1	protein tyrosine phosphatase, non-receptor type 1
(2,5)	Pou3f1	POU domain, class 3, transcription factor 1
(2,5)	Plcb3	phospholipase C, beta 3
(2,5)	Nfatc2ip	nuclear factor of activated T-cells, cytoplasmic, calcineurin-dependent 2 interacting protein
(2,5)	Npas1	neuronal PAS domain protein 1
(2,5)	Mybbp1a	MYB binding protein (P160) 1a
(2,5)	Map2k7	mitogen activated protein kinase kinase 7
(2,5)	Mccc1	methylcrotonoyl-Coenzyme A carboxylase 1 (alpha)
(2,5)	Smad7	MAD homolog 7 (Drosophila)
(2,5)	Ltbp2	latent transforming growth factor beta binding protein 2
(2,5)	Igfbp6	insulin-like growth factor binding protein 6
(2,5)	Hebp1	heme binding protein 1
(2,5)	Hspa1l	heat shock protein 1-like
(2,5)	Gnaq	guanine nucleotide binding protein, alpha q polypeptide
(2,5)	Grb2	growth factor receptor bound protein 2
(2,5)	Gstm2	glutathione S-transferase, mu 2
(2,5)	Flot1	flotillin 1
(2,5)	Flnb	filamin, beta
(2,5)	Fgf5	fibroblast growth factor 5
(2,5)	Ch25h	cholesterol 25-hydroxylase
(2,5)	Ches1	checkpoint suppressor 1

(2,5)	Casp7	caspase 7
(2,5)	Creb1	cAMP responsive element binding protein 1
(2,5)	Bmx	BMX non-receptor tyrosine kinase
(2,5)	Rnpep	arginyl aminopeptidase (aminopeptidase B)
(2,5)	Ass1	argininosuccinate synthetase 1
(2,5)	Ankrd47	ankyrin repeat domain 47
(2,5)	Asb6	ankyrin repeat and SOCS box-containing protein 6
(2,5)	Aoc3	amine oxidase, copper containing 3
(2,5)	Naglu	alpha-N-acetylglucosaminidase (Sanfilippo disease IIIB)
(2,6)	Zswim1	zinc finger, SWIM domain containing 1
(2,6)	Zfp444	zinc finger protein 444
(2,6)	Tnnt3	troponin T3, skeletal, fast
(2,6)	Trim21	tripartite motif protein 21
(2,6)	Tmem11	transmembrane protein 11
(2,6)	Trh	thyrotropin releasing hormone
(2,6)	Snta1	syntrophin, acidic 1
(2,6)	Socs3	suppressor of cytokine signaling 3
(2,6)	Shh	sonic hedgehog
(2,6)	Sstr4	somatostatin receptor 4
(2,6)	Slc26a1	solute carrier family 26 (sulfate transporter), member 1
(2,6)	Slc25a20	solute carrier family 25 (mitochondrial carnitine/acylcarnitine translocase), member 20
(2,6)	Slc22a18	solute carrier family 22 (organic cation transporter), member 18
(2,6)	Smu1	smu-1 suppressor of mec-8 and unc-52 homolog (C. elegans)
(2,6)	Sirt3	sirtuin 3 (silent mating type information regulation 2, homolog) 3 (S. cerevisiae)
(2,6)	Six6	sine oculis-related homeobox 6 homolog (Drosophila)
(2,6)	Serpina3n	serine (or cysteine) peptidase inhibitor, clade A, member 3N
(2,6)	Sema6d	sema domain, transmembrane domain (TM), and cytoplasmic domain, (semaphorin) 6D
(2,6)	Sema4f	sema domain, immunoglobulin domain (Ig), TM domain, and short cytoplasmic domain
(2,6)	Rom1	rod outer segment membrane protein 1
(2,6)	Rbms2	RNA binding motif, single stranded interacting protein 2
(2,6)	Arhgdig	Rho GDP dissociation inhibitor (GDI) gamma
(2,6)	Rxrg	retinoid X receptor gamma
(2,6)	Rax	retina and anterior neural fold homeobox
(2,6)	Rtn2	reticulon 2 (Z-band associated protein)
(2,6)	Rage	renal tumor antigen
(2,6)	Ramp3	receptor (calcitonin) activity modifying protein 3
(2,6)	Rab11b	RAB11B, member RAS oncogene family
(2,6)	Pdk4	pyruvate dehydrogenase kinase, isoenzyme 4
(2,6)	Ptp4a3	protein tyrosine phosphatase 4a3
(2,6)	Ppp3cc	protein phosphatase 3, catalytic subunit, gamma isoform
(2,6)	Prkce	protein kinase C, epsilon
(2,6)	Pcsk2	proprotein convertase subtilisin/kexin type 2
(2,6)	Col2a1	procollagen, type II, alpha 1
(2,6)	Pou2f3	POU domain, class 2, transcription factor 3
(2,6)	Pou2f2	POU domain, class 2, transcription factor 2

(2,6)	Kcnj3	potassium inwardly-rectifying channel, subfamily J, member 3
(2,6)	Plcd1	phospholipase C, delta 1
(2,6)	Pla2g6	phospholipase A2, group VI
(2,6)	Pde6g	phosphodiesterase 6G, cGMP-specific, rod, gamma
(2,6)	Pde1c	phosphodiesterase 1C
(2,6)	Pde1b	phosphodiesterase 1B, Ca2+-calmodulin dependent
(2,6)	Pi4k2a	phosphatidylinositol 4-kinase type 2 alpha
(2,6)	Padi1	peptidyl arginine deiminase, type I
(2,6)	Pdlim4	PDZ and LIM domain 4
(2,6)	Pvalb	parvalbumin
(2,6)	Ppy	Pancreatic polypeptide
(2,6)	Prop1	paired like homeodomain factor 1
(2,6)	Pax4	paired box gene 4
(2,6)	Nr2c2	nuclear receptor subfamily 2, group C, member 2
(2,6)	Nr1i3	nuclear receptor subfamily 1, group I, member 3
(2,6)	Nr1i2	nuclear receptor subfamily 1, group I, member 2
(2,6)	Notch4	Notch gene homolog 4 (Drosophila)
(2,6)	Nkx2-9	NK2 transcription factor related, locus 9 (Drosophila)
(2,6)	Myo7a	myosin VIIa
(2,6)	Mta2	metastasis-associated gene family, member 2
(2,6)	Mt3	metallothionein 3
(2,6)	Matn1	matrilin 1, cartilage matrix protein 1
(2,6)	Mknk1	MAP kinase-interacting serine/threonine kinase 1
(2,6)	Ltk	leukocyte tyrosine kinase
(2,6)	Ksr1	kinase suppressor of ras 1
(2,6)	Kcns1	K+ voltage-gated channel, subfamily S, 1
(2,6)	Jak3	Janus kinase 3
(2,6)	Jag2	jagged 2
(2,6)	Il12rb2	interleukin 12 receptor, beta 2
(2,6)	Irf4	interferon regulatory factor 4
(2,6)	Itih4	inter alpha-trypsin inhibitor, heavy chain 4
(2,6)	Has1	hyaluronan synthase1
(2,6)	Hoxb6	homeo box B6
(2,6)	Hoxa2	homeo box A2
(2,6)	Hdac5	histone deacetylase 5
(2,6)	H2-Ab1	histocompatibility 2, class II antigen A, beta 1
(2,6)	Hmox2	heme oxygenase (decycling) 2
(2,6)	Gng3	guanine nucleotide binding protein (G protein), gamma 3 subunit
(2,6)	Gdf5	growth differentiation factor 5
(2,6)	Gp5	glycoprotein 5 (platelet)
(2,6)	Gcat	glycine C-acetyltransferase (2-amino-3-ketobutyrate-coenzyme A ligase)
(2,6)	Gstm5	glutathione S-transferase, mu 5
(2,6)	Grin1	glutamate receptor, ionotropic, NMDA1 (zeta 1)
(2,6)	Gabra2	gamma-aminobutyric acid (GABA-A) receptor, subunit alpha 2
(2,6)	Galr3	galanin receptor 3

(2,6)	Foxn1	forkhead box N1
(2,6)	Foxj1	forkhead box J1
(2,6)	Fpgs	folylpolyglutamyl synthetase
(2,6)	Fgf18	fibroblast growth factor 18
(2,6)	Fgf12	fibroblast growth factor 12
(2,6)	Fntb	farnesyltransferase, CAAX box, beta
(2,6)	Erf	Ets2 repressor factor
(2,6)	Esrra	estrogen related receptor, alpha
(2,6)	Esr1	estrogen receptor 1 (alpha)
(2,6)	Ephx2	epoxide hydrolase 2, cytoplasmic
(2,6)	Ephx1	epoxide hydrolase 1, microsomal
(2,6)	Eno3	enolase 3, beta muscle
(2,6)	Ezh1	enhancer of zeste homolog 1 (Drosophila)
(2,6)	Edn3	endothelin 3
(2,6)	Ets1	E26 avian leukemia oncogene 1, 5' domain
(2,6)	Ddit4	DNA-damage-inducible transcript 4
(2,6)	Cyp27b1	cytochrome P450, family 27, subfamily b, polypeptide 1
(2,6)	Cdk5r2	cyclin-dependent kinase 5, regulatory subunit 2 (p39)
(2,6)	Cdk5r1	cyclin-dependent kinase 5, regulatory subunit (p35) 1
(2,6)	Cuedc2	CUE domain containing 2
(2,6)	Crhr1	corticotropin releasing hormone receptor 1
(2,6)	Ccs	copper chaperone for superoxide dismutase
(2,6)	Clpb	ClpB caseinolytic peptidase B homolog (E. coli)
(2,6)	Ctrl	chymotrypsin-like
(2,6)	Chrd	chordin
(2,6)	Ccl27	chemokine (C-C motif) ligand 27
(2,6)	Cited1	Cbp/p300-interacting transactivator with Glu/Asp-rich carboxy-terminal domain 1
(2,6)	Crebl1	cAMP responsive element binding protein-like 1
(2,6)	Capn5	calpain 5
(2,6)	Baiap2	brain-specific angiogenesis inhibitor 1-associated protein 2
(2,6)	Bhmt	betaine-homocysteine methyltransferase
(2,6)	Aip	aryl-hydrocarbon receptor-interacting protein
(2,6)	Arnt	aryl hydrocarbon receptor nuclear translocator
(2,6)	Accn1	amiloride-sensitive cation channel 1, neuronal (degenerin)
(2,6)	Apc2	adenomatosis polyposis coli 2
(2,6)	Acp6	acid phosphatase 6, lysophosphatidic
(2,6)	Adam23	a disintegrin and metallopeptidase domain 23
(2,6)	Adam12	a disintegrin and metallopeptidase domain 12 (meltrin alpha)
(3,1)	Tmem40	transmembrane protein 40
(3,1)	Tm4sf1	transmembrane 4 superfamily member 1
(3,1)	Mdm2	transformed mouse 3T3 cell double minute 2
(3,1)	Scap2	src family associated phosphoprotein 2
(3,1)	Slc7a7	solute carrier family 7 (cationic amino acid transporter, y+ system), member 7
(3,1)	Six5	sine oculis-related homeobox 5 homolog (Drosophila)
(3,1)	LOC238836	similar to H3 histone, family 3B

(3,1)	Sgk	serum/glucocorticoid regulated kinase
(3,1)	Stk10	serine/threonine kinase 10
(3,1)	Serpinb6a	serine (or cysteine) peptidase inhibitor, clade B, member 6a
(3,1)	Sqstm1	sequestosome 1
(3,1)	4930504E06Rik	RIKEN cDNA 4930504E06 gene
(3,1)	2700055K07Rik	RIKEN cDNA 2700055K07 gene
(3,1)	Rxra	retinoid X receptor alpha
(3,1)	Ranbp10	RAN binding protein 10
(3,1)	Ptprc	protein tyrosine phosphatase, receptor type, C
(3,1)	Kcnb1	potassium voltage gated channel, Shab-related subfamily, member 1
(3,1)	Pcaf	p300/CBP-associated factor
(3,1)	Ostf1	osteoclast stimulating factor 1
(3,1)	Nfyc	nuclear transcription factor-Y gamma
(3,1)	Nr4a2	nuclear receptor subfamily 4, group A, member 2
(3,1)	Nr4a1	nuclear receptor subfamily 4, group A, member 1
(3,1)	Nupr1	nuclear protein 1
(3,1)	Myo5a	myosin Va
(3,1)	Mpl	myeloproliferative leukemia virus oncogene
(3,1)	Mzf1	myeloid zinc finger 1
(3,1)	Myc	myelocytomatosis oncogene
(3,1)	Max	Max protein
(3,1)	Mxd1	MAX dimerization protein 1
(3,1)	Mkrn1	makorin, ring finger protein, 1
(3,1)	Klf4	Kruppel-like factor 4 (gut)
(3,1)	Klf2	Kruppel-like factor 2 (lung)
(3,1)	Klhl21	kelch-like 21 (Drosophila)
(3,1)	Icam1	intercellular adhesion molecule
(3,1)	Impdh1	inosine 5'-phosphate dehydrogenase 1
(3,1)	Hoxa6	homeo box A6
(3,1)	H2-Ea	histocompatibility 2, class II antigen E alpha
(3,1)	Hck	hemopoietic cell kinase
(3,1)	Hspb8	heat shock protein 8
(3,1)	Gbp2	guanylate nucleotide binding protein 2
(3,1)	Gabarapl2	gamma-aminobutyric acid (GABA-A) receptor-associated protein-like 2
(3,1)	Gprasp1	G protein-coupled receptor associated sorting protein 1
(3,1)	Foxp1	forkhead box P1
(3,1)	Fcer1a	Fc receptor, IgE, high affinity I, alpha polypeptide
(3,1)	Eif1a	eukaryotic translation initiation factor 1A
(3,1)	Eif1	eukaryotic translation initiation factor 1
(3,1)	Ereg	epiregulin
(3,1)	Epha2	Eph receptor A2
(3,1)	Ell2	elongation factor RNA polymerase II 2
(3,1)	Dnase1l3	deoxyribonuclease 1-like 3
(3,1)	Dhrs8	dehydrogenase/reductase (SDR family) member 8
(3,1)	Dcp1a	decapping enzyme

(3,1)	Cyb5r1	cytochrome b5 reductase 1
(3,1)	Cxxc1	CXXC finger 1 (PHD domain)
(3,1)	Commd3	COMM domain containing 3
(3,1)	Cd244	CD244 natural killer cell receptor 2B4
(3,1)	Cd1d1	CD1d1 antigen
(3,1)	Cebpd	CCAAT/enhancer binding protein (C/EBP), delta
(3,1)	Bst1	bone marrow stromal cell antigen 1
(3,1)	Bid	BH3 interacting domain death agonist
(3,1)	Relb	avian reticuloendotheliosis viral (v-rel) oncogene related B
(3,1)	Abcb1a	ATP-binding cassette, sub-family B (MDR/TAP), member 1A
(3,1)	Arntl	aryl hydrocarbon receptor nuclear translocator-like
(3,1)	Acyp2	acylphosphatase 2, muscle type
(3,1)	Anp32b	acidic nuclear phosphoprotein 32 family, member B
(3,4)	Zfp706	zinc finger protein 706
(3,4)	Vamp3	vesicle-associated membrane protein 3
(3,4)	Tpd52l1	tumor protein D52-like 1
(3,4)	Tubb6	tubulin, beta 6
(3,4)	Tagln	transgelin
(3,4)	Tcf20	transcription factor 20
(3,4)	Skil	SKI-like
(3,4)	Sec61g	SEC61, gamma subunit
(3,4)	S100a11	S100 calcium binding protein A11 (calizzarin)
(3,4)	Rhoa	ras homolog gene family, member A
(3,4)	Ppp5c	protein phosphatase 5, catalytic subunit
(3,4)	Pkp3	plakophilin 3
(3,4)	Plcb1	phospholipase C, beta 1
(3,4)	Mpg	N-methylpurine-DNA glycosylase
(3,4)	Nov	nephroblastoma overexpressed gene
(3,4)	Mfap5	microfibrillar associated protein 5
(3,4)	Met	met proto-oncogene
(3,4)	Lsr	lipolysis stimulated lipoprotein receptor
(3,4)	Ltbp4	latent transforming growth factor beta binding protein 4
(3,4)	Lama3	laminin, alpha 3
(3,4)	Kpna4	karyopherin (importin) alpha 4
(3,4)	Hhat	Hedgehog acyltransferase
(3,4)	Hsf2	heat shock factor 2
(3,4)	Grhl2	Grainyhead-like 2 (Drosophila)
(3,4)	G6pdx	glucose-6-phosphate dehydrogenase X-linked
(3,4)	Gbx2	gastrulation brain homeobox 2
(3,4)	Fosl2	fos-like antigen 2
(3,4)	Fkbp1a	FK506 binding protein 1a
(3,4)	Epb4.1l4a	erythrocyte protein band 4.1-like 4a
(3,4)	Dus1l	dihydrouridine synthase 1-like (S. cerevisiae)
(3,4)	Cpt1a	carnitine palmitoyltransferase 1a, liver
(3,4)	Cbr3	carbonyl reductase 3

(3,4)	Atxn1	ataxin 1
(3,4)	Avpr1a	arginine vasopressin receptor 1A
(3,4)	Actn4	actinin alpha 4
(3,4)	Adam19	a disintegrin and metallopeptidase domain 19 (meltrin beta)
(3,5)	Zfp30	zinc finger protein 30
(3,5)	Zfp110	zinc finger protein 110
(3,5)	Zfp106	zinc finger protein 106
(3,5)	Tmem131	transmembrane protein 131
(3,5)	Tm7sf3	transmembrane 7 superfamily member 3
(3,5)	Tle4	transducin-like enhancer of split 4, homolog of Drosophila E(spl)
(3,5)	Tcfap2c	transcription factor AP-2, gamma
(3,5)	Txn1	thioredoxin 1
(3,5)	Tspan5	tetraspanin 5
(3,5)	Socs2	suppressor of cytokine signaling 2
(3,5)	Sfrs6	splicing factor, arginine/serine-rich 6
(3,5)	Scye1	small inducible cytokine subfamily E, member 1
(3,5)	Setdb1	SET domain, bifurcated 1
(3,5)	Rpo1-3	RNA polymerase 1-3
(3,5)	0910001A06Rik	RIKEN cDNA 0910001A06 gene
(3,5)	Rexo2	REX2, RNA exonuclease 2 homolog (S. cerevisiae)
(3,5)	Rbck1	RanBP-type and C3HC4-type zinc finger containing 1
(3,5)	Rab8b	RAB8B, member RAS oncogene family
(3,5)	Ppa2	pyrophosphatase (inorganic) 2
(3,5)	Ptpn2	protein tyrosine phosphatase, non-receptor type 2
(3,5)	Ppp2r5c	protein phosphatase 2, regulatory subunit B (B56), gamma isoform
(3,5)	Kcnk1	potassium channel, subfamily K, member 1
(3,5)	Prps2	phosphoribosyl pyrophosphate synthetase 2
(3,5)	Pik3c3	phosphoinositide-3-kinase, class 3
(3,5)	Olfr90	olfactory receptor 90
(3,5)	Ncoa3	nuclear receptor coactivator 3
(3,5)	Nf2	neurofibromatosis 2
(3,5)	Ngfb	nerve growth factor, beta
(3,5)	Naca	nascent polypeptide-associated complex alpha polypeptide
(3,5)	Map4k4	mitogen-activated protein kinase kinase kinase kinase 4
(3,5)	Mcm3ap	minichromosome maintenance deficient 3 (S. cerevisiae) associated protein
(3,5)	Mme	membrane metallo endopeptidase
(3,5)	Ifrd1	interferon-related developmental regulator 1
(3,5)	Hsd11b2	hydroxysteroid 11-beta dehydrogenase 2
(3,5)	Hoxd3	homeo box D3
(3,5)	Hoxb3	homeo box B3
(3,5)	Hoxa7	homeo box A7
(3,5)	Hdgf	hepatoma-derived growth factor
(3,5)	Hsp110	heat shock protein 110
(3,5)	Gna14	guanine nucleotide binding protein, alpha 14
(3,5)	Ghitm	growth hormone inducible transmembrane protein

(3,5)	Gab1	growth factor receptor bound protein 2-associated protein 1
(3,5)	Gclm	glutamate-cysteine ligase , modifier subunit
(3,5)	Gtf2f2	general transcription factor IIF, polypeptide 2
(3,5)	D6Wsu176e	DNA segment, Chr 6, Wayne State University 176, expressed
(3,5)	D1Pas1	DNA segment, Chr 1, Pasteur Institute 1
(3,5)	Cox7b	cytochrome c oxidase subunit VIIb
(3,5)	Calml4	calmodulin-like 4
(3,5)	Cdh1	cadherin 1
(3,5)	Brd2	bromodomain containing 2
(3,5)	Aldh2	aldehyde dehydrogenase 2, mitochondrial
(3,5)	Atf4	activating transcription factor 4
(3,6)	Wnt2b	wingless related MMTV integration site 2b
(3,6)	Wt1	Wilms tumor homolog
(3,6)	Vav2	Vav2 oncogene
(3,6)	Tcfcp2l1	transcription factor CP2-like 1
(3,6)	Tcp11	t-complex protein 11
(3,6)	Sycp1	synaptonemal complex protein 1
(3,6)	Speg	SPEG complex locus
(3,6)	Slc5a1	solute carrier family 5 (sodium/glucose cotransporter), member 1
(3,6)	Slc20a1	solute carrier family 20, member 1
(3,6)	Shmt1	serine hydroxymethyl transferase 1 (soluble)
(3,6)	S100a6	S100 calcium binding protein A6 (calcyclin)
(3,6)	Rbpsuhl	recombining binding protein suppressor of hairless-like (Drosophila)
(3,6)	Pdk1	pyruvate dehydrogenase kinase, isoenzyme 1
(3,6)	Pcx	pyruvate carboxylase
(3,6)	Ptprn2	protein tyrosine phosphatase, receptor type, N polypeptide 2
(3,6)	Col9a2	procollagen, type IX, alpha 2
(3,6)	Pla2g5	phospholipase A2, group V
(3,6)	Prph1	peripherin 1
(3,6)	Pax5	paired box gene 5
(3,6)	Oprd1	opioid receptor, delta 1
(3,6)	Nr5a2	nuclear receptor subfamily 5, group A, member 2
(3,6)	Nr5a1	nuclear receptor subfamily 5, group A, member 1
(3,6)	Nr0b1	nuclear receptor subfamily 0, group B, member 1
(3,6)	Npy5r	neuropeptide Y receptor Y5
(3,6)	Npy2r	neuropeptide Y receptor Y2
(3,6)	Mylpf	myosin light chain, phosphorylatable, fast skeletal muscle
(3,6)	Msc	musculin
(3,6)	Mt4	metallothionein 4
(3,6)	Mep1b	meprin 1 beta
(3,6)	Mc3r	melanocortin 3 receptor
(3,6)	Ly6g6d	lymphocyte antigen 6 complex, locus G6D
(3,6)	Lif	leukemia inhibitory factor
(3,6)	Lefty1	left right determination factor 1
(3,6)	Krt2	keratin 2

(3,6)	Itgb3	integrin beta 3
(3,6)	Inhbb	inhibin beta-B
(3,6)	Hivep3	human immunodeficiency virus type I enhancer binding protein 3
(3,6)	Hoxa1	homeo box A1
(3,6)	Gadd45g	growth arrest and DNA-damage-inducible 45 gamma
(3,6)	Gcm1	glial cells missing homolog 1 (Drosophila)
(3,6)	Gtf2f1	general transcription factor IIF, polypeptide 1
(3,6)	Gabpa	GA repeat binding protein, alpha
(3,6)	Foxn2	forkhead box N2
(3,6)	Fert2	fer (fms/fps related) protein kinase, testis specific 2
(3,6)	Fancc	Fanconi anemia, complementation group C
(3,6)	Epha3	Eph receptor A3
(3,6)	Enc1	ectodermal-neural cortex 1
(3,6)	Dmrt1	doublesex and mab-3 related transcription factor 1
(3,6)	Ddit3	DNA-damage inducible transcript 3
(3,6)	Cyp2b10	cytochrome P450, family 2, subfamily b, polypeptide 10
(3,6)	C1ql1	complement component 1, q subcomponent-like 1
(3,6)	Commd1	COMM domain containing 1
(3,6)	Cldn11	claudin 11
(3,6)	Cd80	CD80 antigen
(3,6)	Cd19	CD19 antigen
(3,6)	Car3	carbonic anhydrase 3
(3,6)	Camk2d	calcium/calmodulin-dependent protein kinase II, delta
(3,6)	Bckdha	branched chain ketoacid dehydrogenase E1, alpha polypeptide
(3,6)	Akr1b8	aldo-keto reductase family 1, member B8
(3,6)	Arl4a	ADP-ribosylation factor-like 4A
