# Supplementary material for: Modeling Co-Expression across Species for Complex Traits: Insights to the Difference of Human and Mouse Embryonic Stem Cells
Source: PLoS Comput Biol. 2010 Mar 12;6(3):e1000707. doi: 10.1371/journal.pcbi.1000707 (PMC2837392; doi:10.1371/journal.pcbi.1000707)
Supplement: Table S4 — Conserved transcription regulators in human and mouse ES cells. Genes with a & may act as transcriptional repressors or corepressors. (0.01 MB PDF) [file pcbi.1000707.s012.pdf]

Table S4: Conserved transcription regulators in human and mouse ES cells. Genes with a & may act as transcriptional repressors or corepressors.

Function	Gene
Basic transcription machinery	BTF3, TAF1C, RRN3
Sequence dependent transcription factors	OCT4, SOX2, NANOG, UTF1, ZFP64, MTF2, ETV4, POU1F1, POU2F1, HESX1, E2F8[&], TGIF1[&], CTBP2[&]
Chromatin modification	EED[&], PHC1[&], SMARCD1, SUPT4H1[&], SUV39H1, MYST2, RUVBL1
DNA methylation	DNMT1[&], DNMT3B[&]
Histone acetylation	SAP30[&], MYST2
